# Thermochemical properties and life cycle assessment of waste spent coffee grounds

**DOI:** 10.1038/s41598-026-54060-8

**Published:** 2026-05-21

**Authors:** Theoklitos Klitou, Kyriaki Koumenidou, Michalis Menicou, Paris A. Fokaides

**Affiliations:** 1 School of Engineering, Frederick University, Nicosia, Cyprus; 2 Faculty of Civil Engineering and Architecture, Kaunas University of Technology, Kaunas, Lithuania

**Keywords:** Spent coffee grounds, Pelletization, LCA, Waste management, Biomass combustion, Energy science and technology, Engineering, Environmental sciences, Materials science

## Abstract

Spent coffee grounds (SCGs) represent a widely available organic residue with significant potential for energy valorization. This study evaluates the feasibility of converting SCGs into fuel pellets. Thermochemical characterization indicated promising properties for energy recovery and informed the development of a subsequent life cycle assessment (LCA). A cradle-to-gate LCA, performed using the ILCD methodology, assesses four pelletization scenarios: a baseline grid-powered process, sun drying, photovoltaic electricity supply, and their combination.The results identify drying and transportation as the dominant contributors to environmental impacts. The combined sun drying and photovoltaic scenario achieves the lowest impacts across all assessed categories, reducing greenhouse gas emissions and fossil resource depletion by more than 50% compared to the baseline.A sensitivity analysis was conducted on key parameters, including moisture content (25%, 50%—baseline scenario, and 75%), electricity supply based on alternative European grid mixes, and transportation distance for both the collection of SCGs and the distribution of pellets. The analysis highlights the importance of balanced moisture content, the influence of electricity generation profiles, and the benefits of reduced transportation distances on overall environmental performance.A preliminary cost–benefit perspective is also presented to outline key economic considerations, forming the basis for a more detailed techno-economic assessment in future work.

## Introduction

 In today’s fast-paced world, energy powers our everyday lives, but the reliance on fossil fuels is quickly losing its appeal. As technology evolves and the global population grows, energy demands continue to rise, bringing sustainable alternatives into focus. Traditional fuels not only harm the environment but are also depleting at an unsustainable rate. Renewable Energy Sources (RES) have emerged as critical, eco-friendly solutions for the future. Among them, biomass stands out as a particularly promising option, offering the potential to help the EU achieve its environmental targets while simultaneously reducing waste and pollution. This transition marks a significant step toward a cleaner and more sustainable future^1–4^.

Coffee is undoubtedly one of the most widely consumed beverages globally, with an estimated 2.25 billion cups consumed daily^5^. In Cyprus, coffee holds a prominent place in everyday life, deeply embedded in social and cultural traditions. Although the country’s spent coffee ground (SCG) output is modest in comparison to major coffee-consuming nations, the consistent growth in local consumption presents a promising opportunity for valorization. SCGs are increasingly recognized as a valuable secondary resource, contributing to sustainable waste management and circular economy initiatives. By reimagining coffee waste as a renewable input for bioenergy or material applications, Cyprus can adopt innovative, environmentally responsible practices that align with international sustainability trends while leveraging its local consumption habits.

The last five (5) years, 2018 to 2024, the out-of-home volume for roasted coffee in Cyprus grew steadily, increasing from 1.30 million kilograms in 2018 to an expected 1.26 million kilograms in 2024^6^. This upward trend is projected to continue, reaching 1.44 million kilograms by 2029. In the τ table below (Table 1), the per capita consumption of roast coffee from 2018 to July 2024 (actual data) is shown, along with projections through 2029. The figure highlights a decline in 2020, likely due to external factors as the COVID-19 pandemic, followed by a recovery in subsequent years. Consumption is expected to stabilize at around 0.9 kg per capita from 2027 onward. Given the current data and future projections, the valorization of spent coffee grounds (SCGs) in Cyprus presents a substantial and advantageous opportunity^6^.

**Table 1:** Per capita consumption of roast coffee from 2018 to July 2024 (actual data) and projections from 2025 to 2029, measured in kilograms per person per year^6^.

Volume per capita (roast coffee) (Kg)	Year
0.85	2018
0.87	2019
0.67	2020
0.70	2021
0.73	2022
0.76	2023
0.78	2024
0.81	2025
0.83	2026
0.86	2027
0.89	2028
0.89	2029

Beyond their market potential, Spent Coffee Grounds (SCGs) are increasingly catching the eye of eco-conscious innovators. With their impressive calorific value, SCGs are being repurposed as a sustainable energy source, making waves in biofuel production and green energy solutions. The circular economy thrives through the valorization of spent coffee grounds (SCGs), which bridges the gap between coffee culture and renewable energy. Repurposing SCGs offers a sustainable and economically viable solution to reducing waste while harnessing their latent potential. This approach aligns with the UN Sustainable Development Goals #6 (Clean Water and Sanitation), #7 (Affordable and Clean Energy), and #12 (Responsible Consumption and Production), contributing to a greener and more responsible future^5^ .

**Fig. 1:**
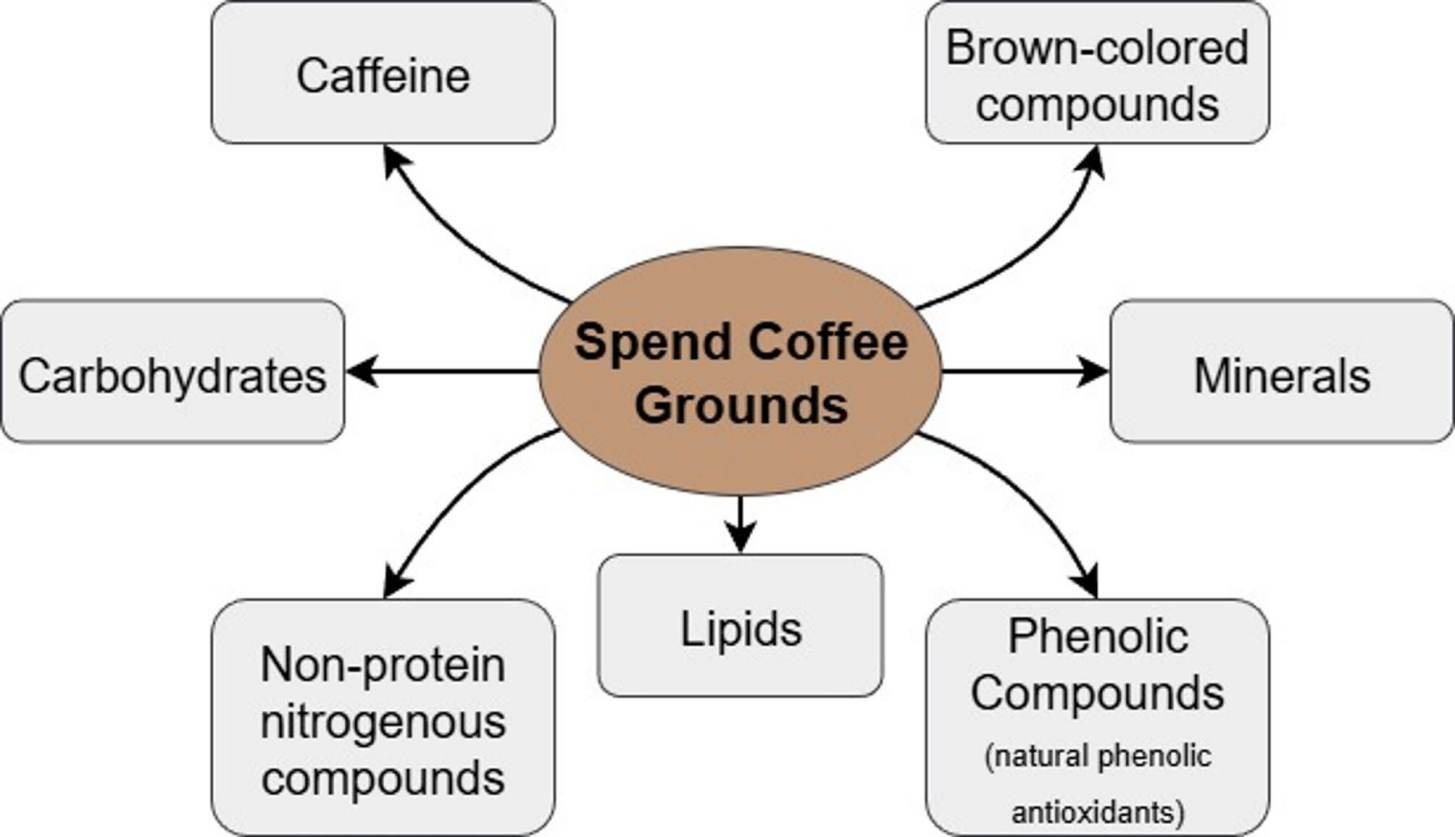
SCGs valorization ways.

As comprehensively reviewed by Campos-Vega et al. (2015), early innovation efforts prior to 2005 were largely centered on the selective extraction of individual high-value constituents from spent coffee grounds (SCGs), including oils, flavor compounds, terpenes, and alcohols. In more recent years, research has increasingly shifted toward bioenergy production and biorefinery concepts, positioning SCGs as a versatile feedstock for fuels, chemicals, and materials. Despite this expanded research landscape, the review highlights that practical implementations have remained largely fragmented, with most valorization pathways targeting single components rather than the holistic utilization of the SCG matrix. Consequently, integrated fractionation strategies capable of systematically exploiting carbohydrates, lipids, phenolic compounds, nitrogenous compounds, minerals, caffeine, and Maillard-derived brown compounds within a unified industrial framework are still scarce. As illustrated in Fig. 1, SCGs constitute a chemically rich and multifunctional biomass, underscoring the significant yet underexploited potential for fully integrated, multi-product valorization approaches beyond conventional first-generation waste management routes^.^

Spent coffee grounds (SCGs) are an abundant agro-industrial biomass residue generated during the brewing and processing of coffee. According to Campos-Vega et al.^7^, SCGs exhibit a complex chemical composition that includes carbohydrates, lipids, proteins, phenolic compounds, minerals, caffeine, and Maillard-derived brown compounds, supporting their classification as a high-potential lignocellulosic biomass feedstock. Unlike dedicated energy crops, SCGs are continuously available and low-cost, as they arise from an existing food supply chain, making them particularly attractive within circular economy frameworks. Their compositional diversity enables multiple valorization pathways, including bioenergy production and the recovery of value-added compounds, while simultaneously addressing waste management and environmental sustainability challenges.

The circular potential is evident in the repurposing of SCGs, as a source of energy, turning a waste product into a valuable resource. However, it is important to consider the efficiency of these biofuel processes and their overall environmental impact.

Despite the significant expansion of research in recent years, there remains a notable deficiency in publications addressing fuel pellets and their combustion in heating applications. This gap becomes more pronounced when considering studies specifically focused on fuel pellets made from spent coffee grounds (SCGs) and the processes involved in their production. Although the publication rate in this niche area is inconsistent, the overall trend suggests a gradual increase in interest. Notably, only one published paper to date has focused on the life cycle assessment (LCA) of fuel pellets made from SCGs^2^.

This paper investigates the potential for the sustainable reuse of spent coffee grounds (SCGs) as fuel pellets, examining both the characteristics of SCGs and their combustion properties, as well as the life cycle assessment (LCA) of the pelletization process.

## Theoretical background/literature review

**Fig. 2:**
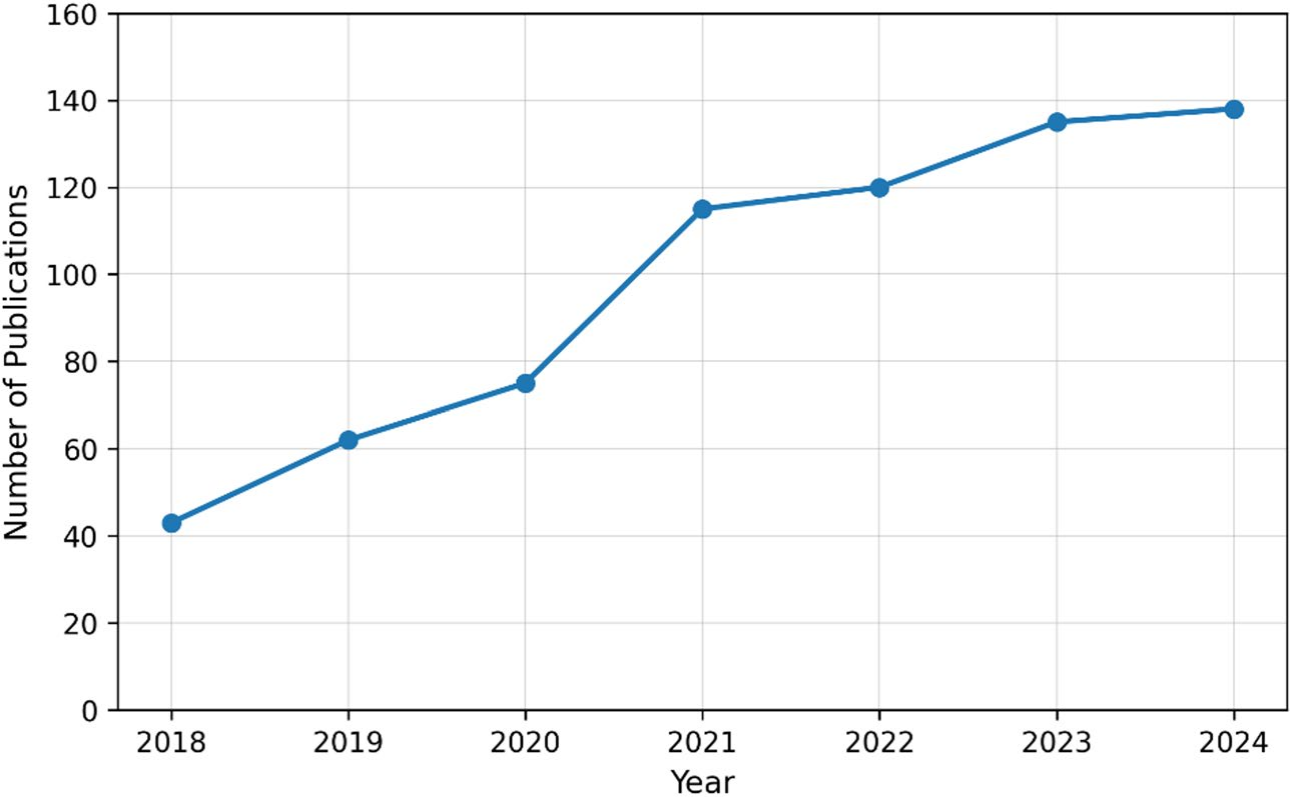
Number of publications concerning SCGs, the last five (5) years.

Research on spent coffee grounds (SCGs) and their potential applications is increasingly gaining attraction. As is shown in the Fig. 2, over the past five years, the number of publications on this topic has steadily risen, reflecting a growing interest in their diverse uses. The production of these studies has yet to stabilize, indicating a dynamic field ripe for innovation.

**Fig. 3:**
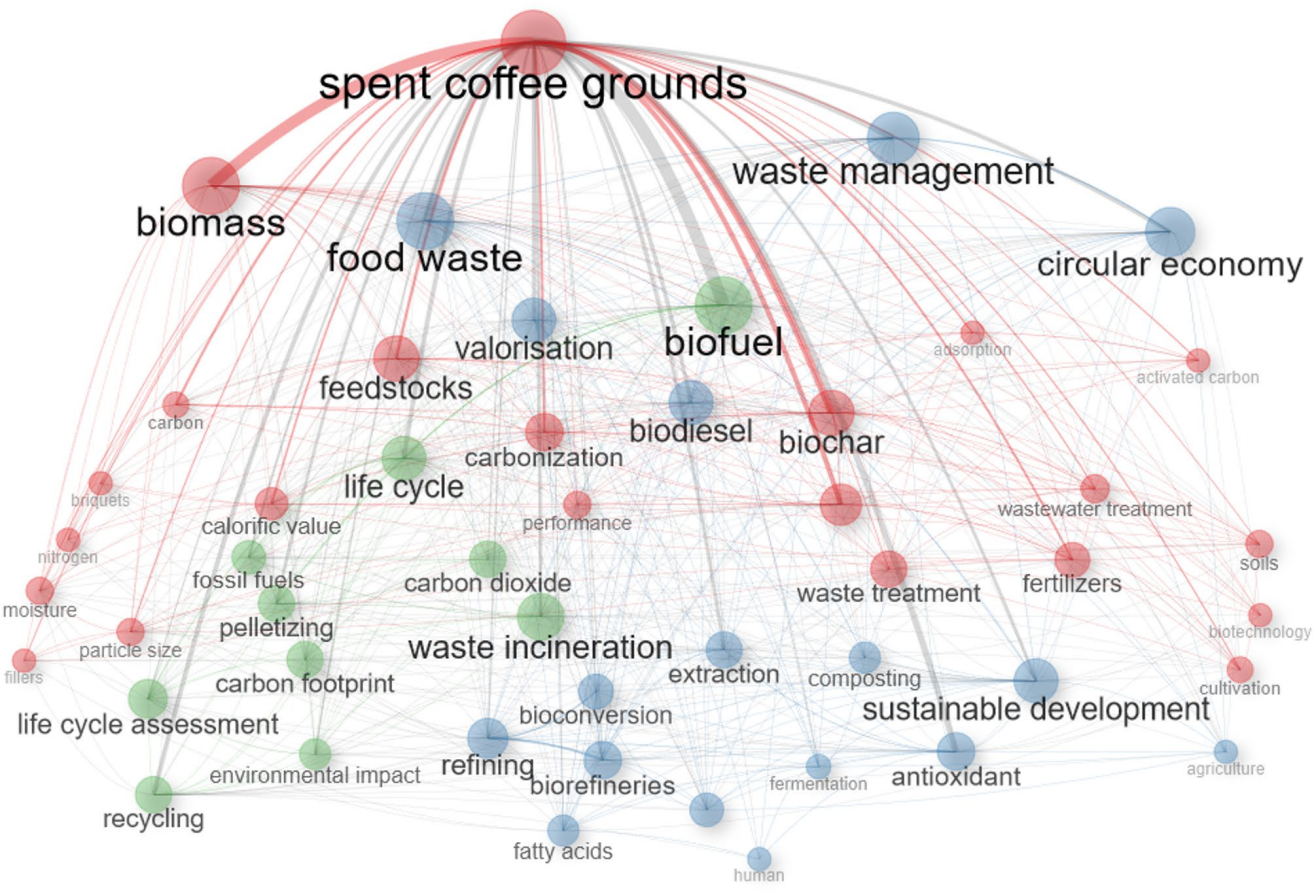
Thematic network map on spent coffee grounds for the last five (5) years (2018–2024).

Research in this area weaves through a rich tapestry of disciplines, all converging around a shared focus on environmental impact and sustainability. A thematic network has been crafted (Fig. 3) to spotlight recent explorations (2018–2024) into Spent Coffee Grounds (SCG), mapping out the tightly interlinked fields that gravitate toward this topic. As depicted in the figure, SCG appears at the heart of research themes like Biomass, Waste Management, Sustainable Development, and the Circular Economy, revealing an eco-conscious shift that reimagines “waste” as a resource in the journey toward a more sustainable, circular future.

Spent Coffee Grounds (SCGs) find themselves intricately connected to a constellation of themes, including biomass, feedstock, food waste, biochar, biodiesel, waste management, and waste incineration—signaling a nuanced exploration into the valorization of coffee waste. The biofuel nexus links with emerging themes that underscore a heightened interest in sustainable bioenergy solutions, such as waste management, life cycle assessment, and environmental impact. Waste Management and Sustainable Development further align with the circular economy, forming a cohesive narrative around resource regeneration.

The thematic map reveals a vision for SCGs as transformative assets in a circular bioeconomy. These processes not only mitigate climate change but also reduce landfill dependency, cut carbon emissions, minimize resource consumption, and advance sustainable development. Yet, for SCGs to fully realize their potential as eco-friendly assets, further research and practical implementation remain essential.

From 1977, with the first ever published study of the possible reuse of SCGs, until the 2000s, studies were rare and fluctuating, in contrast with the last years where the interest is growing and the number of publications is going up^8^.

**Fig. 4:**
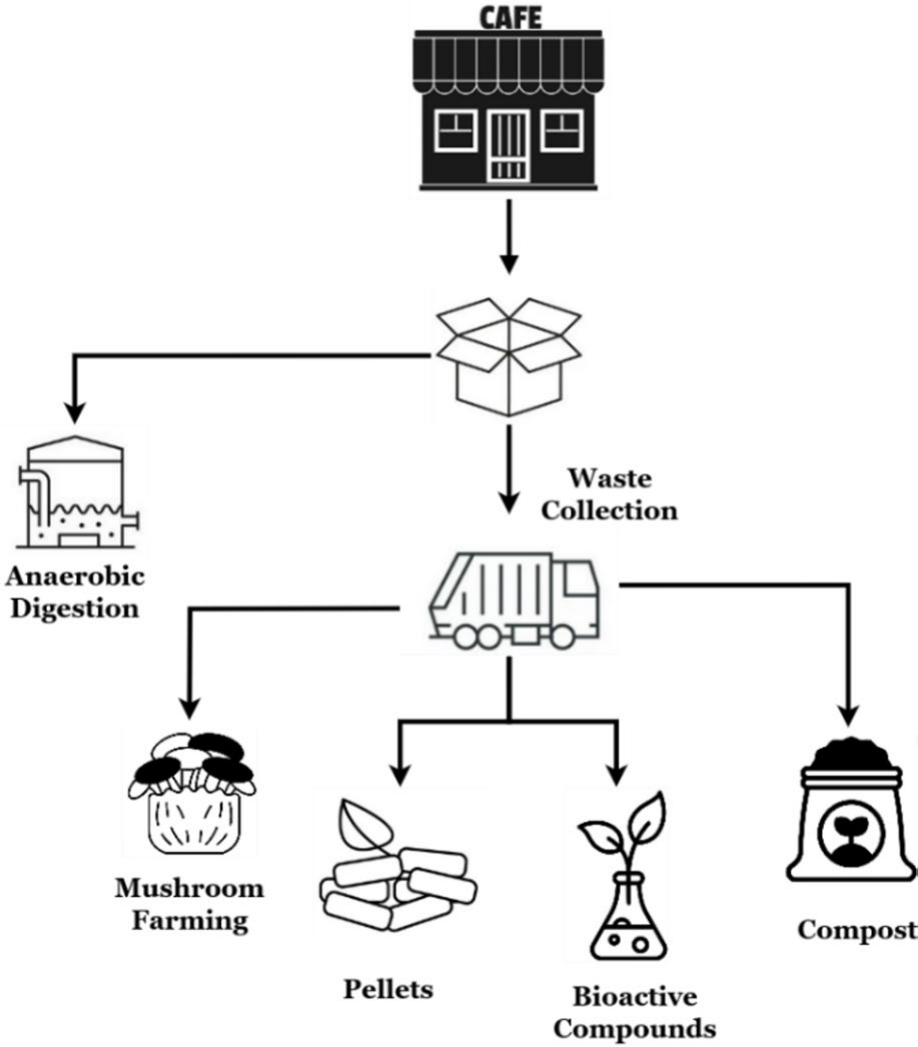
End uses for spent coffee grounds (SCGs).

Recent advancements in technologies for valorizing spent coffee grounds (SCGs) encompass processes such as anaerobic digestion, pyrolysis, liquefaction, gasification, oil extraction, fermentation, and various other methods aimed at producing value-added products, including compost, adsorbents, antioxidants, and nutrients [5]. The potential end uses of SCGs are shown in the following Fig. 4.

SCGs are a rich source of bioactive compounds with antioxidant, antimicrobial, and anti-inflammatory properties, valuable in various industries (Zuorro and Lavecchia et al.^9^ . Anaerobic digestion of SCGs produces methane-rich biogas and nutrient-rich fertilizer, though expanding feedstock to include all food waste from cafes and restaurants could enhance its efficacy. A 2023 study (Vitale et al., 2023) shows that Expresso Coffee By-products can be used as natural fertilizers^10^.

Through the application of innovative recycling techniques, SCGs can function as sustainable biobased fertilizers to address the growing demands of an expanding global population. However, when generated in substantial quantities, SCGs present significant environmental concerns due to the increased oxygen demand required for their decomposition and the presence of harmful compounds such as caffeine, tannins, and polyphenols. As a result, SCGs are not widely applicable for large-scale composting and are often disposed of in landfills, leading to considerable economic and environmental implications^5^. Conversely, bioenergy derived from SCGs has garnered significant attention in recent years due to their non-seasonal availability and widespread distribution, offering a promising alternative for sustainable energy applications.

### Mushroom growth medium

Additionally, studies (Carrasco-Cabrera et al., 2019) showed that Spent Coffee Grounds (SCG) serve as an effective substrate for cultivating edible mushrooms, particularly oyster mushrooms, offering comparable nutritional quality and yields to traditional substrates, though large-scale implementation remains limited^11^. Also, studies have shown that using SCG as a growth substrate provides mushrooms with the same nutritional quality and yields as mushrooms grown on substrates currently utilized by the mushroom industry, such as straw^12^. Furthermore, the mushrooms generated have neither caffeine nor tannins, and the fungus is expected to partially break down the toxins in the SCG^13^. SCG seems to be an excellent substrate for mycelia development but not for mushroom growth in one research^14^. Oyster mushrooms tend to be the best type for growing on SCG, as they are reasonably easy to grow and more resistant to changing environmental conditions^15^.

### Bioactive compounds

Coffee beans are a rich tapestry of bioactive compounds, including phenolic compounds, melanoidins, diterpenes, xanthines, and vitamin precursors. Each contributes uniquely to coffee’s potential health benefits. For instance, chlorogenic acids—the predominant element in coffee seeds—have been associated with reduced risks of atherosclerosis, diabetes, and several cancers^16,17^. Melanoidins, formed in the roasting process, exhibit an impressive array of health-promoting effects: they have been shown to inhibit bacterial growth in the colon, possess anti-inflammatory and antiglycative properties, and may even help thwart tumor growth and metastasis^18,19^. Though conventional brewing only partially extracts these compounds, spent coffee grounds (SCG) remain a compelling, underutilized source of bioactive elements. Studies have unearthed a wealth of antioxidants within SCG, boasting a high phenolic content that rivals conventional sources^20^. Yet, despite mounting evidence supporting SCG’s value in the food and pharmaceutical sectors, a standardized, scalable method for its utilization has yet to take root in industry.

### Fuel pellets

Turning raw solid biomass material into pellets has a high potential for energy usage. Spent coffee grounds (SCGs) hold significant potential as an energy source through their conversion into fuel pellets and various biofuels. Because of the high efficiency of the combustion, the coffee pellets have a high calorific value content with a low amount of non-usable material remaining (ash) and negligible carbon monoxide emissions. The pellets, on the other hand, have a high moisture content, low bulk density, and mechanical resilience. The densification process, which compresses biomass into pellets with higher energy density, offers a promising way to enhance the energy potential of SCG. The global market for fuel pellets (FPs) is expanding as these densified solids serve as a renewable substitute for coal in electricity and power generation, as well as residential and district heating. By transforming SCGs into fuel pellets, we can contribute to climate change mitigation, reduce landfill waste, lower carbon emissions, and decrease freshwater eutrophication. This study aims to assess the total environmental impact of the pelletization process.

The combustion characteristics of spent coffee grounds (SCGs), repurposed as biofuel pellets, reveal a promising alternative energy source with a blend of efficiency and environmental responsibility. During testing^21^, conducted in accordance with the CYS EN 303-5:2012 standard^14^, SCG pellets demonstrated impressive combustion efficiency, maintaining an average of 89%. CO emissions, which initially registered at 1166.3 ppm, steadily declined to 317.3 ppm as the flame temperature increased, a testament to the robust combustion dynamics facilitated by high temperatures and optimized oxygen flow. Flue gas temperatures rose consistently, underscoring the pellets’ stable heat output.

These coffee-derived pellets not only met but exceeded European emissions standards for Class 3 boilers, proving their viability as a cleaner, more sustainable energy source^22^. Their low carbon monoxide emissions, coupled with high flame efficiency and minimal ash residue, mark them as an ideal candidate for environmentally conscious heating solutions. While aspects such as bulk density and moisture content present opportunities for refinement, the overall performance of SCG pellets highlights their potential in the world of biofuel—a sustainable energy choice with a touch of artisanal charm (The combustion).

Spent Coffee Grounds (SCGs) can be processed into a variety of biofuels, including biohydrogen, biobutanol, biodiesel, bio-oil, bioethanol, biogas, and hydrocarbon fuels. Technologies such as pyrolysis demonstrate promise in producing high-quality biofuels and biochar, thereby addressing the fuel crisis and contributing to climate change mitigation efforts. However, further research is required to ensure that SCG pellets comply with standards for density, mechanical stability, hydrophobicity, and energy content. Additionally, the environmental sustainability of biodiesel production is variable; while it is generally viewed as more sustainable than fossil fuels, its advantages may be compromised by the choice of feedstock and production methods, particularly when deforested land or significant chemical inputs are utilized^22–24^. 

## Methodology

### Materials

Coffee grounds from an espresso machine sample given by Coffee Island were used in this investigation. The sampling was performed according to the procedures described in EN 14780:2017^25^. Solid biofuels were placed in air-tight plastic containers. Each sample was labelled with a unique identification number and the date/ time of sampling before being transferred to the laboratory.

### Analysis

In this section, a comprehensive account of the tests conducted to characterize spent coffee grounds, is provided, accompanied by detailed explanations of the techniques employed for each test. The conducted tests are listed below:

- Moisture content.
- Ash content.
- Calorific value.
- C, H, N content.

#### Moisture content determination

The moisture content of the prepared general analysis samples was determined according to the procedure described in EN 18134-3:2015^26^. The samples were placed in the drying furnace (SNOL 20/300) and dried at 105 °C in air atmosphere to achieve constancy in mass. The moisture content was then calculated from the recorded mass loss of the sample using an analytical balance (KERN ABT 220-5DM). The following procedure is demonstrated in Moisture content measurement of solid biofuels 2015.

#### Ash content determination

During the ash content analysis of the samples, the ash content was determined using a high-temperature laboratory furnace (SNOL 4/1100) and KERN ABT 220-5DM analytical balance, following the standardized procedure outlined in EN 18122:2015^27^. The results are reported on a dry basis, considering the calculated moisture content of the samples.

#### Calorific value measurement

The calorific value of the obtained sub-samples was estimated according to EN 18125:2017^28^, Calorific value measurement of solid biofuels using bomb calorimetry 2015, using a Parr 1341 Oxygen Bomb Calorimeter. A calibration of the calorimeter took place prior to the experimental runs using certified benzoic acid pellets, with known calorific value.

#### C, H, N content measurement

The total carbon (C), Hydrogen (H) and Nitrogen (N) content of the samples, was determined according to the procedure described in EN 16948:2015^29^. The tests were conducted using a Perkin Elmer 2400 Series II CHN analyser, which was calibrated prior to the measurements using appropriate substances, i.e., acetanilide, as indicated by the manufacturer. The total C, H, and N content in dry basis was calculated by considering the moisture content of the sample. The following procedure is demonstrated in Elemental analysis of Solid Biofuels 2015.

### Life cycle assessment

The Life Cycle Assessment (LCA) consists of four essential phases. Phase 1: Scope and Definition outlines the goals and boundaries of the assessment. Phase 2: Life Cycle Inventory collects data on the inputs and outputs of the system. Phase 3: Impact Assessment evaluates the environmental impacts based on the inventory data. Finally, Phase 4: Interpretation connects the findings to the original objectives, ensuring the results offer meaningful insights into sustainability.

The objective of this LCA study is to evaluate the environmental implications of the pelleting process in Cyprus, which utilizes spent coffee grounds to produce pellets for residential heating. The entire biomass chain has been prioritized, from the transportation of waste from its creation site to the transfer of manufactured pellets to sales destinations. Spent coffee grounds refer to coffee grounds after they have been used; they are the waste product of the espresso coffee extraction process. The system’s primary role is to produce EN 17225-6:2021 (International Organization for Standardization, 2021) certified non-woody pellets for non-industrial applications.

#### System boundaries

**Fig. 5:**
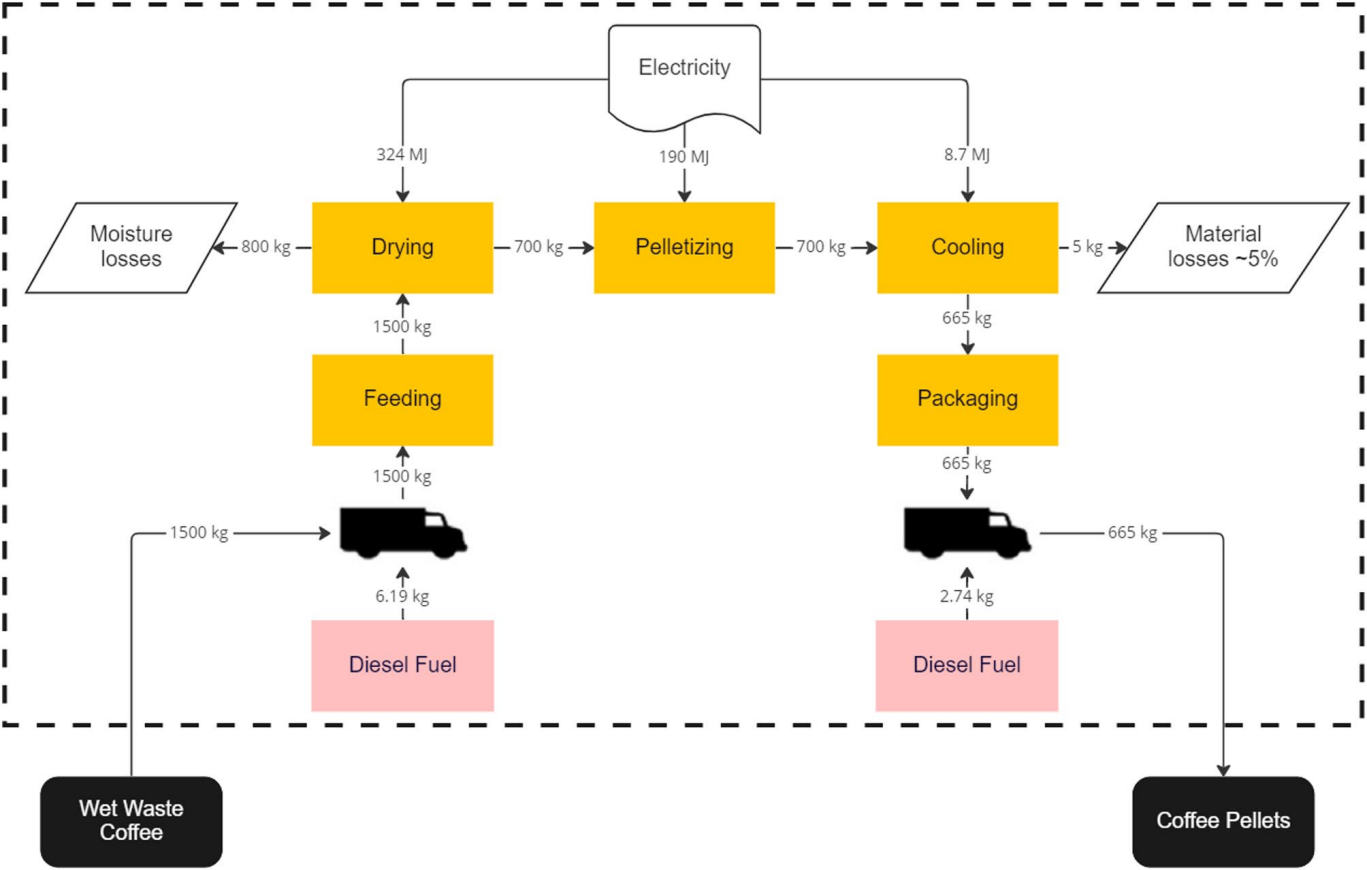
LCA analysis of the SCG pelletization process^31^.

Figure 5 shows a schematic representation of the stages of the biomass pelleting process chain that are included in the scope of the study: transportation of wet waste from the production points to the central management center, feeding, drying, pelleting, cooling, packaging, and distribution of pellets from the management centers back to sale points of the final products. The use of materials and energy for the construction of the employed machinery and infrastructure is excluded from the system boundaries due to its minimal contribution to the overall impact^30^.

#### Impact categories

The Sphera LCA software^32^, as well as the ISO 14040:2006 + A1:2020^33^ and ISO 14044:2006 + A1:2018 + A2:2020^34^ standards, were utilized to implement the LCA in this study. In this work, the International Reference Life Cycle Data System (ILCD) guidelines methodology is applied to execute the life cycle impact assessment (LCIA). The ILCD has released ‘Recommendations for Life Cycle Impact Assessment in the European context’ which chooses the methodology which has been evaluated as the best within the impact category (ILCD handbook recommendations for Life Cycle Impact Assessment 2011)^35^. In this paper, the ReCiPe 2016 v1.1 method was chosen to address the environmental impact categories for this pelletization process.

ReCiPe 2016 v1.1 is a harmonized life cycle impact assessment (LCIA) method that translates elementary flows from a Life Cycle Inventory (LCI) into a limited set of environmental impact scores using characterization factors, and it is designed in a way that provides a comprehensive impact assessment at both midpoint and endpoint levels^36^. It encompasses a broad spectrum of environmental impact categories, addressing issues relevant to human health, ecosystem quality, and resource scarcity^36^.

**Table 2:** ReCiPe 2016 v1.1 environmental impact categories.

ReCiPe 2016 v1.1 impact category (Unit)
Climate change (kg CO₂ eq.)
Climate change, biogenic (kg CO₂ eq.)
Fine particulate matter formation (kg PM₂.₅ eq.)
Fossil resource scarcity (kg oil eq.)
Water consumption (m³)
Freshwater ecotoxicity (kg 1,4-DCB eq.)
Freshwater eutrophication (kg P eq.)
Human carcinogenic toxicity (kg 1,4-DCB eq.)
Human non-carcinogenic toxicity (kg 1,4-DCB eq.)
Ionising radiation (kBq Co-60 eq to air)
Land use (m²a crop eq.)
Marine ecotoxicity (kg 1,4-DCB eq.)
Marine eutrophication (kg N eq.)
Mineral resource scarcity (kg Cu eq.)
Photochemical ozone formation, terrestrial ecosystems (kg NOx eq.)
Photochemical ozone formation, human health (kg NOx eq.)
Ozone depletion (kg CFC-11 eq.)
Terrestrial acidification (kg SO₂ eq.)
Terrestrial ecotoxicity

The choice of specific impact categories for reporting, such as Climate Change, Fine Particulate Matter Formation, Fossil Depletion, Freshwater Eutrophication, Human Toxicity (cancer and non-cancer), Land Use, and Terrestrial Acidification, is often guided by their widespread environmental significance, direct relevance to human and ecosystem health, and their frequent prioritization in scientific publications and policy discussions^37,38^. These categories help identify the most environmentally friendly option/scenario by ensuring a holistic evaluation of environmental performance, which is crucial for informed decision-making in various applications, from food production to waste management and construction^31,39^.

I. Climate Change (Kg CO2 eq.): This category quantifies the contribution of greenhouse gas emissions to global warming, expressed in kilograms of carbon dioxide equivalents.
II. Fine Particulate Matter Formation (Kg PM2.5 eq.): This indicator assesses the potential for the formation of fine particulate matter in the atmosphere, which can negatively affect human respiratory health, measured in kilograms of PM2.5 equivalents.
III. Fossil Depletion (Kg oil eq.): This category represents the depletion of fossil resources due to their extraction and consumption, typically measured in kilograms of oil equivalents.
IV. Freshwater Eutrophication (Kg P eq.): This category evaluates the potential for nutrient enrichment in freshwater systems, leading to excessive algal growth and oxygen depletion, expressed in kilograms of phosphorus equivalents.
V. Human Toxicity, cancer (Kg 1,4-DB eq.): This category assesses the potential for substances to cause carcinogenic effects on human health, quantified in kilograms of 1,4-dichlorobenzene equivalents.
VI. Human Toxicity, non-cancer (Kg 1,4-DB eq.): This category evaluates the potential for substances to cause non-carcinogenic adverse effects on human health, also quantified in kilograms of 1,4-dichlorobenzene equivalents.
VII. Land Use (Annual crop eq.yr): This category measures the impact of land occupation and transformation, often expressed in annual crop equivalents per year, reflecting the agricultural productivity lost or altered.
VIII. Terrestrial Acidification (Kg SO2 eq.): This indicator assesses the potential for emissions to contribute to soil and water acidification, which can harm terrestrial ecosystems, measured in kilograms of sulfur dioxide equivalents.

#### Alternative scenarios

The Life Cycle Assessment (LCA) was initially conducted on the existing palletization process. Following the analysis and evaluation of the preliminary results, the subprocesses with the greatest environmental impact were identified.

Based on these findings, a series of hypothetical scenarios was developed in which one or more parameters were modified, and the environmental burden of each scenario was calculated.

Initially, four different energy supply scenarios were used to evaluate the manufacturing process’s energy consumption. Next, the moisture content of the spent coffee grounds and the electricity use, considering grid mixes from four European regions, were analyzed, as described below.

##### Energy consumption

The three selected and assessed scenarios in addition to the current one are presented in the table below (Table 2).

**Table 3:** Description of the four pelletization scenarios assessed in the Life Cycle Assessment.

Baseline scenario		The pelletization process is carried out using electricity obtained from the grid provided by a state-owned supplier
Scenario 1: sun drying		In the pelletization process, the drying of coffee waste is carried out using solar radiation, while the remaining processes use electricity from the grid provided by a state-owned supplier
Scenario 2: photovoltaics		The entire pelletization process is carried out using energy from photovoltaic panels
Scenario 3: sun drying and photovoltaics		The entire process, except for the drying of coffee residues, is carried out using photovoltaic panels. The drying process is powered by solar radiation

The LCA results for each scenario are discussed in the following section, where a comparative analysis identifying the most environmentally promising solution is presented.

#### Sensitivity analysis

##### Moisture content

The moisture content of the Spent Coffee Grounds (SCGs) was defined as a variable parameter in the analysis. A baseline scenario was set at 50% moisture, based on experimental measurements. Two additional scenarios were considered in the framework of a sensitivity analysis of the process: a low-moisture case at 25% and a high-moisture case at 75%. The functional unit was fixed at 1 tonne of produced coffee pellets; therefore, the quantity of SCGs required varies depending on the moisture content. As the moisture increases, a larger mass of raw material is needed, while lower moisture results in a smaller required input mass. The process most affected by this variation is drying, and the associated electricity consumption is calculated based on the mass of the input material, which is directly determined by its moisture content.

##### Electricity, alternative grid

Electricity supply was treated as a variable parameter by examining different grid mixes. The baseline scenario used Cyprus’s (CY) electricity grid, while other scenarios represented various European regions, selecting specific countries as representative examples.

The electricity supply scenarios were defined as follows:

- Baseline scenario – Cyprus (CY).
- Central and Eastern Europe – Poland (PL).
- Northern Europe – Sweden (SE).
- Southern Europe – Greece (GR).
- Western Europe – France (FR).

The selected countries were used as representative cases for their respective regions.

##### Transportation distance

Transportation distance was also treated as a variable parameter. The distance travelled by the truck for the collection of spent coffee grounds and the distribution of coffee pellets was subjected to a sensitivity analysis. A baseline scenario of 100 km was defined, along with two alternative scenarios at 80 km and 120 km, representing a ± 20% variation.

#### Cost-benefit analysis

A preliminary techno-economic analysis (TEA) was conducted to provide an initial assessment of the economic performance of the proposed process. The analysis considers the main cost components, including capital expenditures (CAPEX) related to equipment, installation, and infrastructure, as well as operational expenditures (OPEX), such as electricity consumption, transportation, labor, and maintenance. The evaluation is based on simplified assumptions appropriate for an early-stage analysis, without the application of a detailed discounted cash flow model. his approach allows for the identification of the main cost drivers and provides an indicative understanding of the economic implications of the system. A comprehensive techno-economic analysis, including detailed financial modeling and levelized cost indicators, is beyond the scope of the present study and is planned for a subsequent stage of the research.

## Results and discussion

### Thermochemical properties

All analyses were conducted twice, and the average values were reported. Table 3 displays the results of the sample analyses alongside two references from the literature. Reference 1^40^ presents the findings of a mixture of Coffee arabica spent coffee grounds naturally dried in the sun, with a moisture content reaching 8.22%. Reference 2^41^ provides the results of 100% Arabica spent coffee grounds of varying origins, with an average moisture content of approximately 52%, dried in a well-ventilated area.

**Table 4:** Reference specifications for pellets.

	Unit	EN standard	Sample			References	
			1	2	Average	1[9]	2[10]
Moisture content,* M*_*ad*_	[w%]	EN ISO 18134-1:2015	54.21	53.37	53.79	Dried 8.22	52
Ash,* A*_*d*_	[w%]	EN ISO 18122:2015	2.23	3.33	2.80	1.59	1.47
Net Calorific value,* q*_*p, net, d*_	[MJ/kg]	EN ISO 18125:2017	20.75	21.19	20.97	19.74	21.26
Carbon content,* C*_*d*_	[w%]	EN ISO 16948:2015	47.81	46.06	46.94	50.26	56.73
Hydrogen content,* H*_*d*_	[w%]	EN ISO 16948:2015	6.31	6.30	6.31	6.29	7.11
Nitrogen content,* N*_*d*_	[w%]	EN ISO 16948:2015	0.46	2.41	1.44	2.21	2.83
Oxygen (by diff.)*, Od*	[w%]	–	45.42	45.23	45.31	31.37	33.19

• ad: as determined basis.

• d: dry basis.

• p, net, d: constant pressure, net, dry.

The standard EN ISO 17225-6:2021 specifies the minimum standards for non-woody pellets.

The moisture content of the tested samples was calculated (i.e., on an as-determined basis, Mar) to be greater than 12 w%, exceeding the permitted limits specified in Table 4. Due to the significant moisture content retained in the coffee, this exceeded the allowable limit. However, it is feasible to allow the used coffee grounds to naturally dry in the sun, achieving a moisture mass share of 12% or less.

As for the ash content of the tested samples, the findings study revealed that the sample’s ash content meets the standards of the EN ISO 17225-6:2021 standard, which specifies a maximum allowable value of 10% on a dry basis. The ash level of pellets produced from biomass feedstock mixes should not exceed 10% by weight.

The net calorific value of the spent coffee grounds sample is acceptable, ranging from 20.75 to 21.19 MJ/kg, and exceeds the minimum standard of 14.5 MJ/kg specified in Table 4.

In terms of elemental analysis, the results, showed that the Nitrogen content level of sample 1 was within the permitted limits shown in Table 4, on contrast with the second sample (sample 2) that was not. The average Nitrogen content of the sample was 1.44 w%, which is lower than the maximum allowable value of 2.0 w%.

**Table 5:** Reference specifications for SCG pellets.

Class	Unit	Non-woody pellets EN ISO 17225-6	
	–	A	B
Moisture content, M^a^_ar_	(w%)	≤ 12	≤ 15
Nitrogen content, N_d_	(w%)	≤ 1.5	≤ 2.0
Ash, A_d_	(w%)	≤ 6.0	≤ 10.0
Net Calorific value, q_p, net, d_	(MJ/kg)	≥ 14.5	≥ 14.5
^a^Wet basis			

### Environmental aspect – life cycle assessment

#### Scope and system boundaries

Following the definition of the system boundaries, the Life Cycle Inventory (LCI) was compiled. It includes detailed information on the transportation of Spent Coffee Grounds (SCG) to the processing facility, the associated energy and mass balances throughout the pelletization process, and the subsequent distribution of the produced coffee pellets to commercial outlets. The system boundaries of this study follow a cradle-to-gate approach, encompassing the collection and transportation of SCG, the drying, grinding, and compression stages involved in pellet production, as well as the transport of the final product to the point of sale. The analysis excludes the use phase of the pellets and their end-of-life treatment, as these lie beyond the defined scope of the current assessment.

#### Life cycle inventory (LCI) – data collection

Table 5 below shows the data utilized for the LCA implementation for this study. The information was gathered via empirical investigations, machinery manufacturers, or was derived from the Sphera LCA database.

Table 5 summarizes the inventory data used for the LCA in this study. Data were obtained from empirical measurements, machinery manufacturers’ specifications, and the Sphera LCA database. The functional unit of the study is defined as 1000 kg (1 tn) of produced coffee pellets. This unit serves as the reference basis for all calculations, ensuring consistency when comparing environmental and economic results across scenarios.

##### Transportation from coffee shops to management centre

**Fig. 6:**
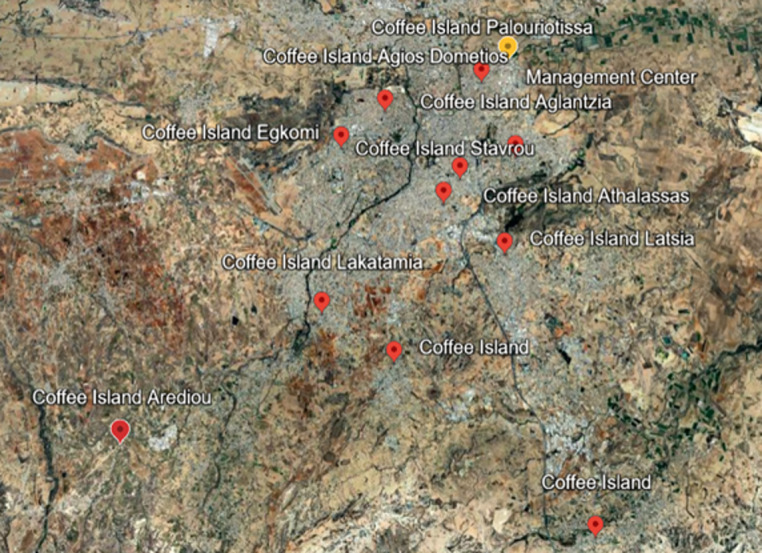
SCG collection points.

The study models the collection of Spent Coffee Grounds (SCGs, 2014 Kg) from Coffee Island coffee shops in the Nicosia district. The management center, located at Frederick University premises in Nicosia, is defined as the starting and ending point of the collection tour. The total travel distance for the collection tour is estimated at 100 km, representing the distance the truck travels to collect the coffee grounds from the selected shops. Figure 6 provides an indicative map where the collection points and the management center are marked.

The same coffee shops are assumed to serve as the final points of sale for the produced pellets (i.e. 1000 kg) after processing; therefore, the same travel distance and route are assumed for pellet distribution in the model.

##### Means of transportation and fuel consumption

Transportation to and from the management center is modeled using a diesel-fueled truck. Diesel consumption is calculated using an existing process from the Sphera LCA database for a Euro V t truck. This process is parameterized by the total distance travelled and the transported mass and returns the corresponding diesel consumption per tour. The truck is assumed to return empty to the management center.

As mentioned in our study Environmental evaluation of biomass pelleting using life cycle assessment (2016)^42^, the values regarding the total fuel consumption for the investigated scenarios were obtained using the transportation distance of predefined collection locations to the plant (GoogleMAPS data) and by utilizing an existing process in the Sphera LCA database for a Euro V truck (Table 6; Fig. 5).

**Table 6:** Life cycle inventory (LCI).

Process and inputs	Value	Units description	Data source
Transport from coffee shops to the management centre	2014	Kg of SCGs	
Diesel	13.36	Kg calculated considering the cargo weight and real distance	Sphera LCA database
Transportation distance	100	km	Google maps
Feeding mass	2014	Kg of SCGs entering the system	
Drying mass loss	1007	Kg	
Drying grid electricity	435.1	MJ	Empirical Data
Pelleting grid electricity	273.4	MJ	Empirical Data
Cooling and screening mass loss	7	Kg	Empirical Data
Cooling grid electricity	12.5	MJ	Empirical Data
Transport from management centre to sale points (i.e. coffee shops)	1000	kg	
Transportation distance	100	km	Google maps
Diesel	6.64	kg	Sphera LCA database

#### Impact assessment

The environmental impact assessment was conducted using Sphera LCA software, applying the ILCD methodology and following ISO 14,040 and 14,044 standards. The analysis focused on eight midpoint impact categories: Climate Change (Kg CO2 eq.), Fine Particulate Matter Formation (Kg PM2.5 eq.), Fossil Depletion (Kg oil eq.), Freshwater Eutrophication (Kg P eq.), Human Toxicity, cancer (Kg 1,4-DB eq.), Human Toxicity, non-cancer (Kg 1,4-DB eq.), Land use (Annual crop eq.yr), Terrestrial Acidification (Kg SO2 eq.).

Results for the baseline scenario (1000 kg of SCG pellets produced) indicate that Climate Change was 252.50 kg CO₂eq, with the drying phase contributing the largest share due to high electricity consumption. Fine Particular Matter Formation was equal to 0.305 Kg PM2.5 eq., and the fossil depletion was 69.396 Kg oil eq, primarily linked to energy and transport inputs. The human toxicity, cancerous and non-cancerous, was equal to 1.952 and 17.599, respectively. In addition, the Freshwater eutrophication and the Terrestrial acidification were 4.83E-04 kg P eq.and 0.961 kg SO2 eq. lastly the Land use was equal to 6.946 annual crop eq. yr. The results are summarized in the Table 7 below.

**Table 7:** ILCD recommendations LCA results for the selected impact categories of the baseline scenario**.**

Env. impact category	Value
Climate change (Kg CO2 eq.)	252.50
Fine particulate matter formation (Kg PM2.5 eq.)	0.302
Fossil depletion (Kg oil eq.)	69.396
Freshwater eutrophication (Kg P eq.)	4.83E-04
Human toxicity, cancer (Kg 1,4-DB eq.)	1.952
Human toxicity, non-cancer (Kg 1,4-DB eq.)	17.599
Land use (annual crop eq.yr)	6.940
Terrestrial acidification (Kg SO2 eq.).	0.961

Table 8 presents the key emissions associated with the pelletization process. Among them, airborne pollutants such as carbon dioxide (CO₂), sulfur dioxide (SO₂), and nitrogen oxides (NOₓ) emerged as the primary contributors across most impact categories. The drying and transportation stages were identified as the most environmentally intensive, highlighting the critical need for energy-efficient interventions. These results provide the rationale for the alternative mitigation scenarios examined in Sect.  3.2.4.

**Table 8:** Data of emissions.

Coffee pellets	
*Inputs*	
Wet waste coffee (kg)	2014
Non-renewable energy resources (MJ)	69.76
Material resources in total	20611.29
Non-renewable elements	0.1229
Non-renewable resources	14.77
Renewable resources	20596.40
*Outputs*	
Coffee pellets ((kg)	1000
*Emissions*	
Emissions to air (kg))	4637
Inorganic emissions to air (kg)	3785
Carbon monoxide (CO) (kg)	0.1126
Carbon dioxide (CO2) (kg)	223.1
Nitrogen oxides (NOx) (kg)	0.2544
Sulphur dioxide (SO2) (kg)	0.7019
Particles to air (kg)	0.02964
Emissions to fresh water (kg)	17319.67
Emissions to sea water (kg)	43.45
Emissions to agricultural soil (kg)	5.20E-5
Inorganic emissions to agricultural soil (kg)	0.0011

The initial results indicate that drying coffee residues collected from retail stores requires the most energy and, consequently, has the greatest environmental impact. In response, the research team developed three alternative scenarios aimed at mitigating this impact as much as possible. The three selected and examined alternatives are presented in Table 2. In addition, the research team evaluated other pelletization-related parameters to assess their influence on environmental impact.

#### Life cycle assessment results

##### Energy consumption

The Life Cycle Assessment of all examined scenarios is presented in the following graphs. Regarding the first Analysis, the Energy Consumption analysis, 8 graphs are presented and each one corresponds to a specific impact category.

Each graph presents the selected impact parameter as a function of the individual processes involved in the pelletization of spent coffee grounds, across the four scenarios previously described. Accompanying each graph is a table that summarizes the corresponding numerical values and reports the relative contribution of each process to the overall environmental impact.

**Fig. 7:**
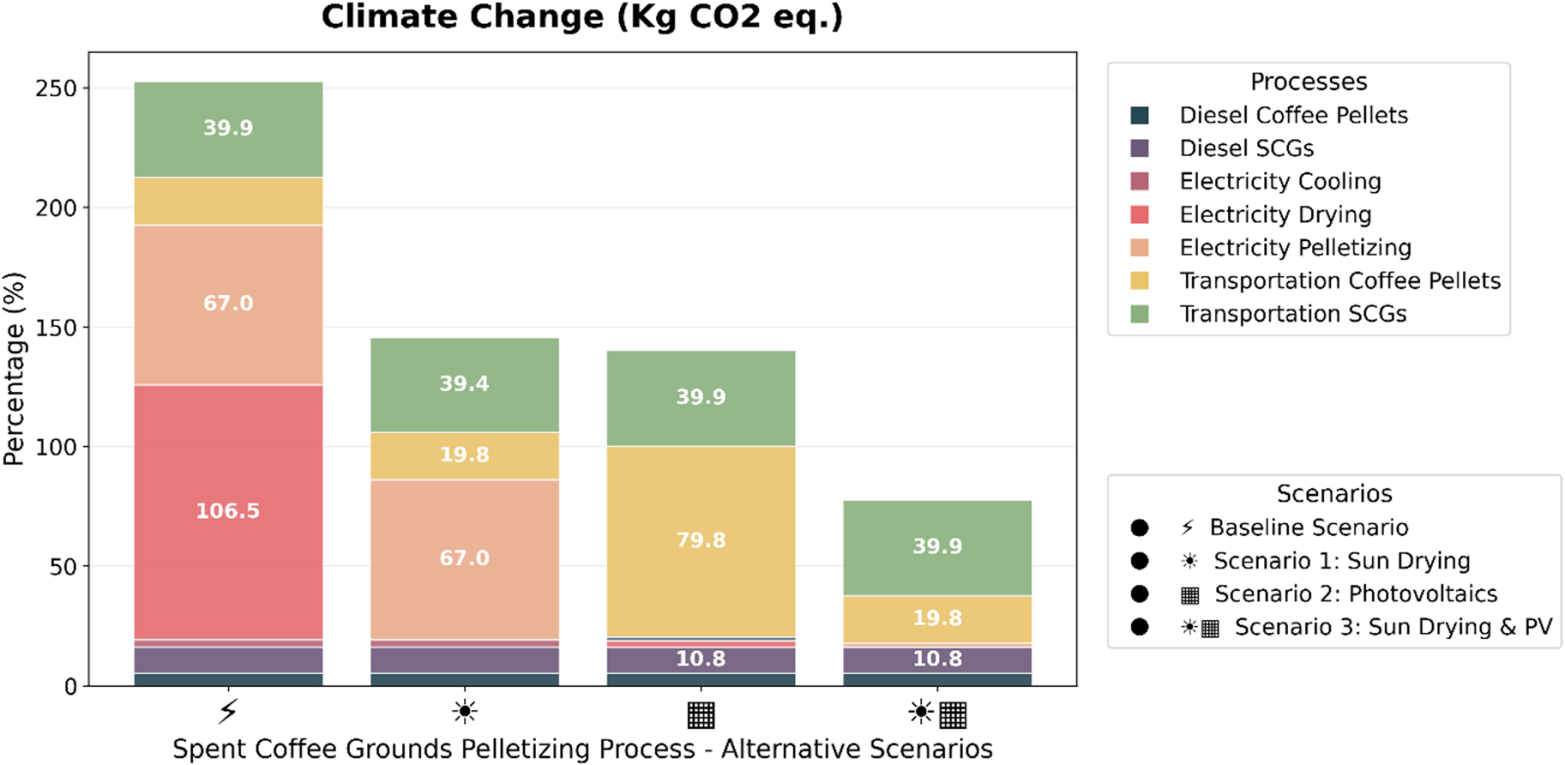
Climate change across alternative scenarios for the pelletization of spent coffee grounds.

The bar chart (Fig. 7), illustrates the Climate Change, expressed in kilograms of CO₂ equivalent (kg CO₂ eq.), associated with the pelletization process of spent coffee grounds (SCGs) under four alternative scenarios. Each bar is segmented to reflect the contribution of individual subprocesses, including transportation, drying, cooling, and pelletizing, to the total Climate Change.

Among the examined cases, the Baseline Scenario demonstrates the highest Climate Change, primarily due to the intensive use of electricity for drying and pelletization, and Diesel for transportation and distribution of the final product, back to the coffee shops. In contrast, Scenario 1 (Sun Drying) significantly lowers emissions by eliminating the electricity demand associated with the drying phase. Scenario 2 (Photovoltaics) achieves further reductions by substituting conventional electricity with renewable energy for drying cooling and pelletizing. The most favorable outcome is observed in Scenario 3 (Sun Drying & Photovoltaics), where the combined use of passive drying and photovoltaic energy yields the lowest overall Climate Change.

**Fig. 8:**
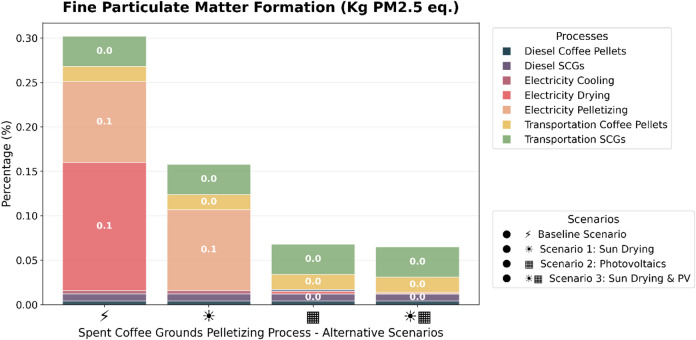
Fine particulate matter formation across alternative scenarios for the pelletization of spent coffee grounds.

Figure 8 presents the Fine Particulate Matter Formation impact, expressed in kg PM2.5 eq., associated with the pelletization of spent coffee grounds (SCGs) across four alternative scenarios, where a similar trend is observed. The Baseline Scenario exhibits the highest values, largely attributed to electricity consumption for drying and pelletizing processes, along with contributions from diesel-based transportation. Scenario 1 achieves a noticeable reduction through the elimination of electrically powered drying, thereby removing a key emission source. Scenario 2 further decreases impacts by substituting fossil-based electricity with photovoltaic energy across the main process stages. The lowest fine particulate matter formation is recorded in Scenario 3, confirming that the combined application of sun drying and photovoltaics offers the most environmentally favorable outcome for this impact category.

**Fig. 9:**
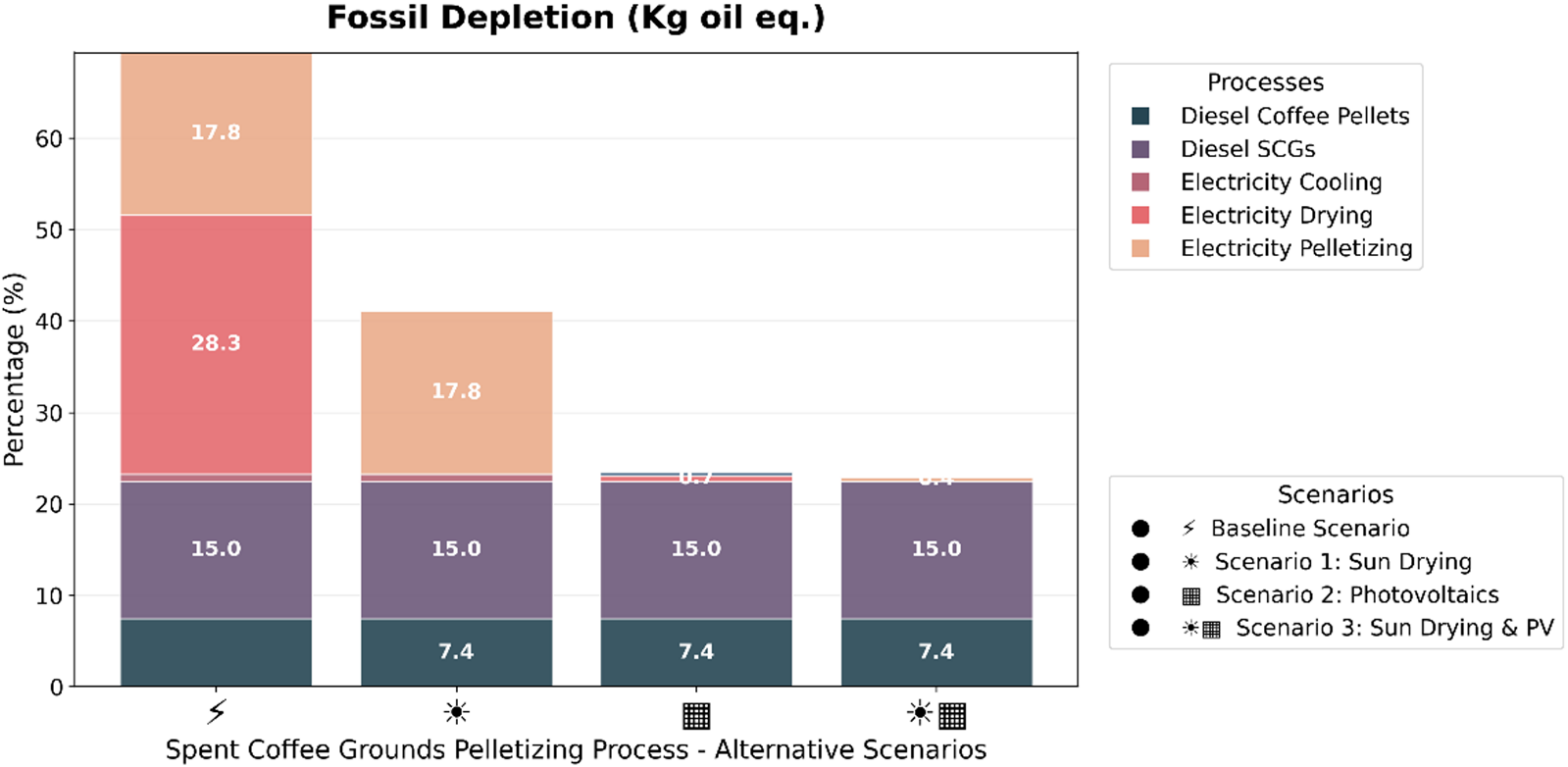
Fossil depletion across alternative scenarios for the pelletization of spent coffee grounds.

Figure 9 presents the Fossil Depletion impact, expressed in Kg oil eq., associated with the pelletization of spent coffee grounds (SCGs) across four alternative scenarios. The Baseline Scenario records the highest fossil depletion, primarily driven by the intensive use of grid electricity in the drying and pelletizing stages, in combination with diesel consumption for transportation of spent coffee grounds. In Scenario 1, a clear reduction is observed due to the removal of electricity demand for drying, which lowers dependence on fossil-based energy inputs. Scenario 2 further mitigates fossil resource use by replacing conventional electricity with photovoltaic energy across the main process operations. The lowest fossil depletion is achieved in Scenario 3, indicating that the integration of sun drying with photovoltaic energy provides the most effective strategy for minimizing reliance on fossil resources within the system.

**Fig. 10:**
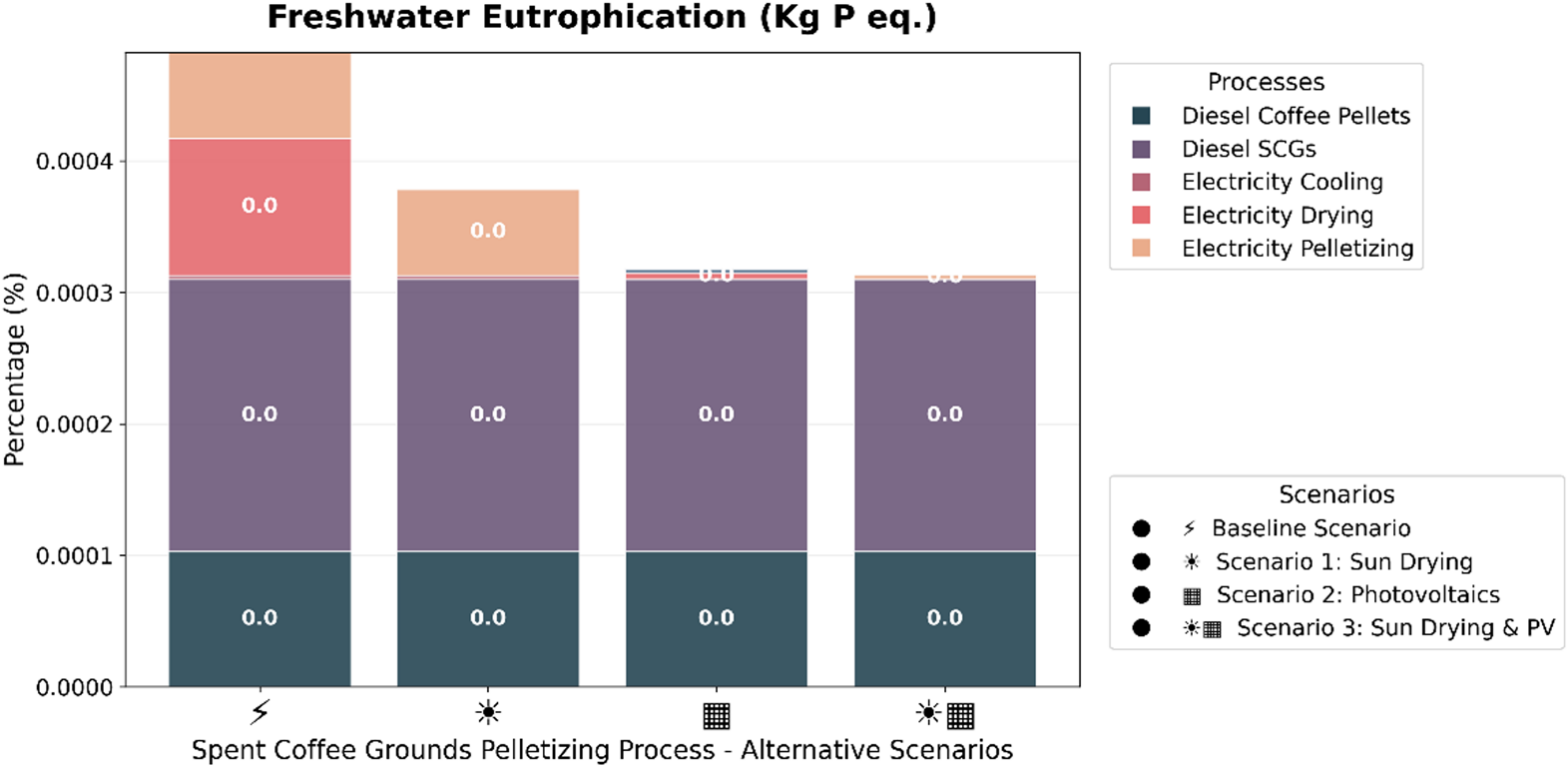
Freshwater eutrophication across alternative scenarios for the pelletization of spent coffee grounds.

Figure 10 presents the Freshwater Eutrophication impact, expressed in Kg P eq., associated with the pelletization of spent coffee grounds (SCGs) across four alternative scenarios. The results for Freshwater Eutrophication further confirm the environmental benefits of the alternative scenarios. The Baseline Scenario shows the highest ecotoxicity values, mainly due to electricity consumption for drying and diesel emissions from transportation and distribution processes. Scenario 1 demonstrates a reduction in overall impact by eliminating electrically driven drying, while Scenario 2 benefits from the substitution of conventional electricity with photovoltaics. As with the previous indicators, Scenario 3 records the lowest ecotoxicity levels, reinforcing the effectiveness of integrating sun drying and renewable energy sources in mitigating environmental burdens.

**Fig. 11:**
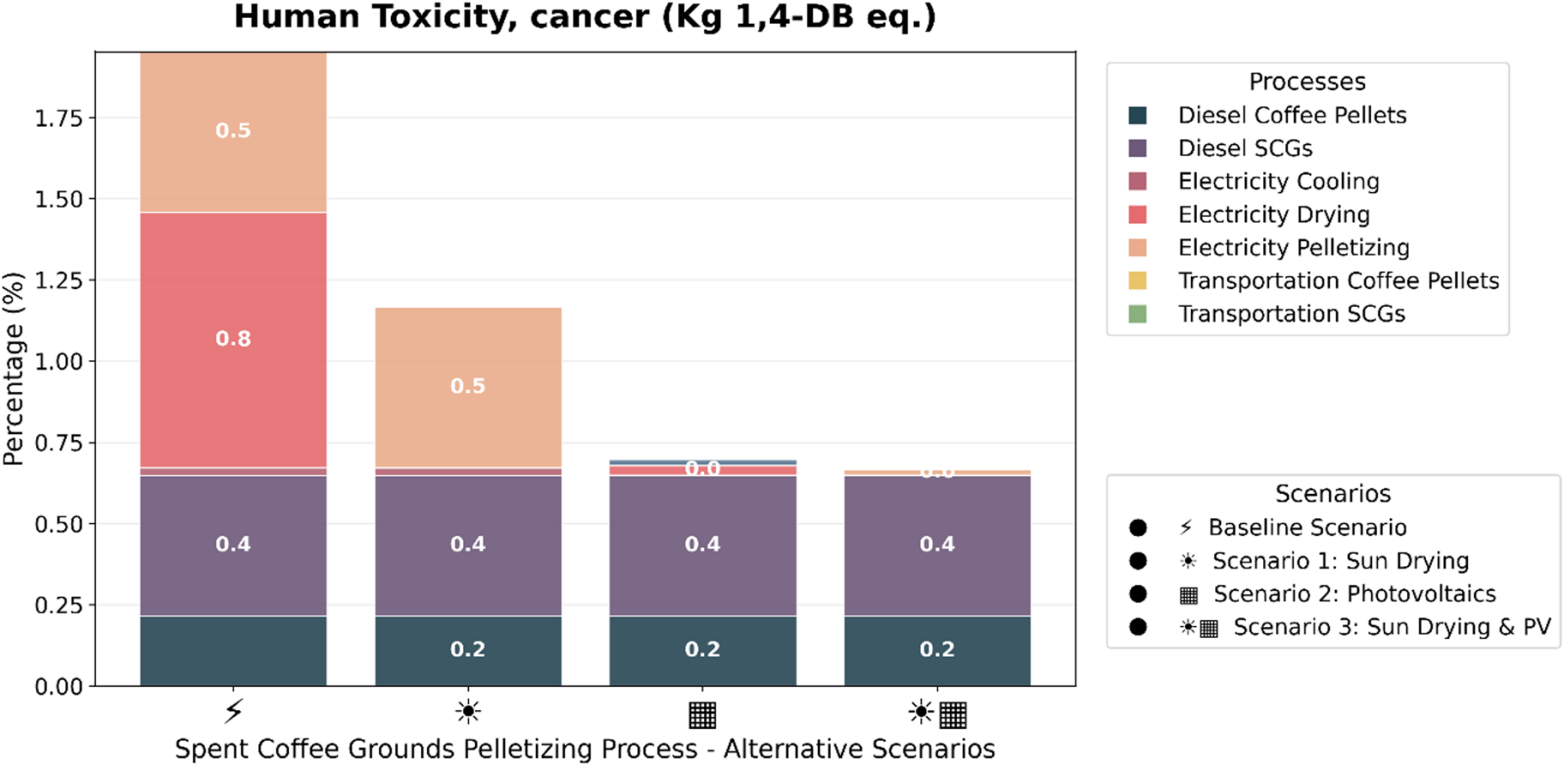
Human toxicity, cancer across alternative scenarios for the pelletization of spent coffee grounds.

Figure 11 presents the Human Toxicity cancer impact, expressed in Kg 1,4-DB eq., associated with the pelletization of spent coffee grounds (SCGs) across the four scenarios The Baseline Scenario records the highest human toxicity (cancer) impact, primarily driven by electricity consumption in the drying and pelletizing processes, along with contributions from diesel use during transportation of spent coffee grounds. In Scenario 1, a reduction is observed as the elimination of electrically powered drying decreases the associated emissions. Scenario 2 further reduces impacts through the substitution of conventional electricity with photovoltaic energy across the main process stages. The lowest human toxicity (cancer) impact is achieved in Scenario 3, indicating that the combined application of sun drying and photovoltaic energy provides the most effective strategy for minimizing health-related environmental burdens.

**Fig. 12:**
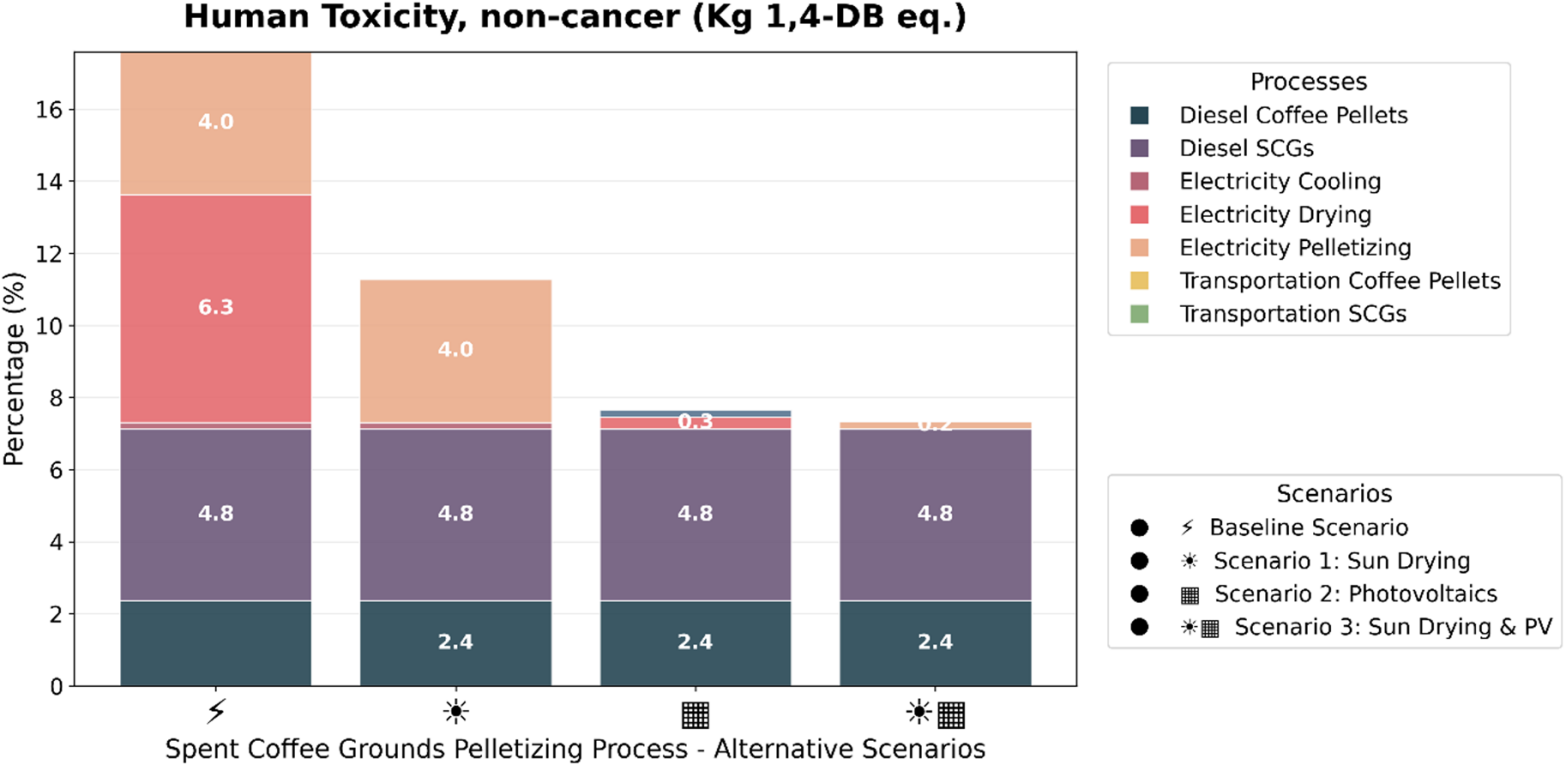
Human toxicity, non-cancer across scenarios for the pelletization of spent coffee grounds.

Figure 12 presents the Human Toxicity non-cancer impact, expressed in Kg 1,4-DB eq., associated with the pelletization of spent coffee grounds (SCGs) across the four scenarios. The Baseline Scenario records the highest human toxicity (non-cancer) impact, mainly associated with electricity consumption in the drying and pelletizing stages, as well as diesel use during transportation for spent coffee grounds. In Scenario 1, a reduction is observed due to the elimination of electricity demand for drying, which lowers the overall emission burden. Scenario 2 further decreases impacts through the substitution of conventional electricity with photovoltaic energy across the main process operations. The lowest human toxicity (non-cancer) impact is achieved in Scenario 3, indicating that the combined use of sun drying and photovoltaic energy represents the most effective approach for reducing non-carcinogenic health impacts within the system.

**Fig. 13:**
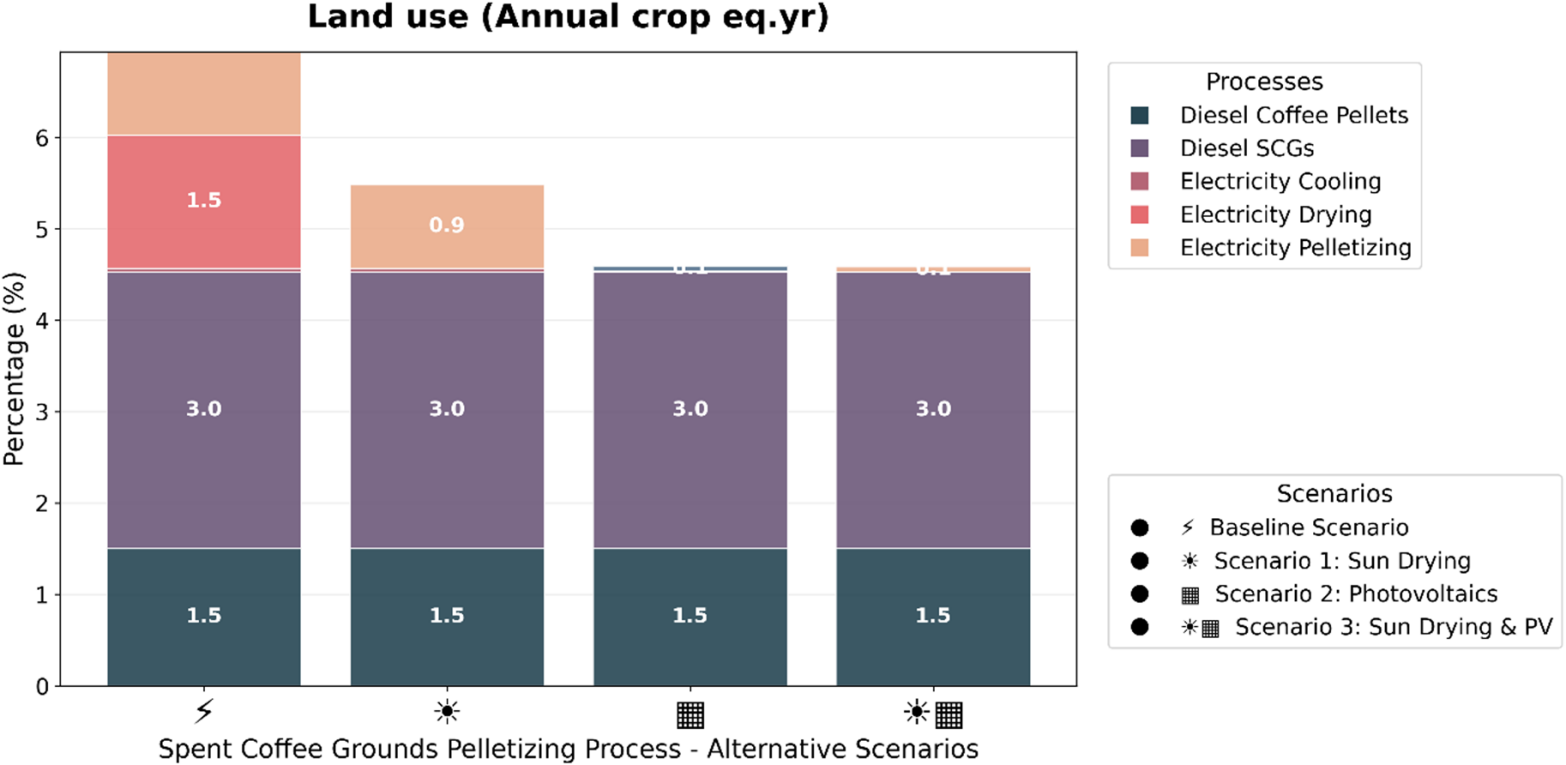
Human toxicity, non-cancer across scenarios for the pelletization of spent coffee grounds.

Figure 13 presents the Human Toxicity non-cancer impact, expressed in Annual crop eq.yr., associated with the pelletization of spent coffee grounds (SCGs) across the four scenarios. The Baseline Scenario records the highest land use impact, primarily driven by electricity consumption in the drying and pelletizing stages. In Scenario 1, a reduction is observed due to the elimination of electrically powered drying, which decreases the overall resource demand. Scenario 2 further limits land use impacts through the substitution of conventional electricity with photovoltaic energy across the main process operations. The lowest land use is achieved in Scenario 3, indicating that the combined application of sun drying and photovoltaic energy provides the most effective approach for minimizing land-related environmental burdens.

**Fig. 14:**
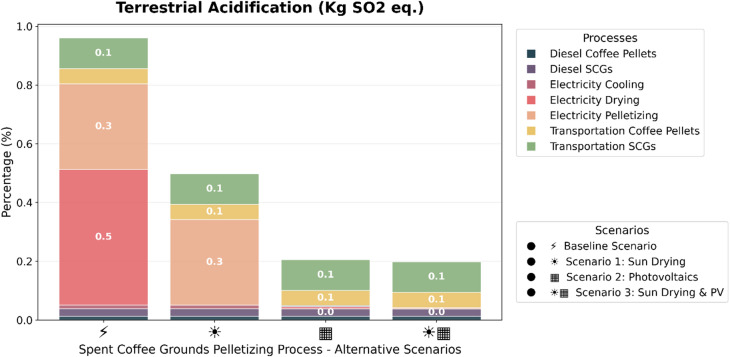
Terrestrial acidification across scenarios for the pelletization of spent coffee grounds.

Figure 14 presents the Terrestrial Acidification impact, expressed in Kg SO2 eq., associated with the pelletization of spent coffee grounds (SCGs) across the four scenarios The Baseline Scenario records the highest terrestrial acidification impact, primarily driven by electricity consumption in the drying and pelletizing stages, along with contributions from diesel-based transportation of the final coffee pellets. In Scenario 1, a clear reduction is observed due to the elimination of electrically powered drying, which significantly lowers the overall emission burden. Scenario 2 further reduces acidification impacts by substituting conventional electricity with photovoltaic energy across the main process operations. The lowest terrestrial acidification is achieved in Scenario 3, indicating that the combined application of sun drying and photovoltaic energy provides the most effective strategy for minimizing acidifying emissions within the system.

The comparative analysis across all environmental impact categories confirms a consistent pattern in the contribution of individual processes within the SCG pelletization system. In the baseline scenario, the drying phase accounts for the highest share of impacts, reaching up to 42.18% of total Climate Change Impact, 47.68% Fine Particulate Matter Impact, 40.83% Fossil Depletion, 40.32 and 35.92% for Human Toxicity, cancer, and non-cancer, respectively, and 48.07% for Terrestrial Acidification. Regarding the rest of the environmental impact categories, Freshwater eutrophication and land use, the process with the highest impact is the Diesel consumption for the transportation of the spent coffee grounds, from the coffee shops to the pelletization unit, with a percentage of 42.90 and 21.64% respectively.

The introduction of sun drying in Scenario 1 eliminates the drying burden and results in a reduction of total Climate Change while Scenario 2, which replaces conventional electricity with photovoltaics, significantly reduces also the emissions associated with pelletizing and cooling. Scenario 3, combining sun drying and photovoltaic systems, consistently achieves the lowest impact scores across all categories, lowering all the examined impact categories. Overall, the results underline the environmental benefits of integrating passive drying and renewable electricity, with percentage-based impact breakdowns offering strong justification for the proposed mitigation scenarios.

##### Sensitivity analysis

The analysis of the other parameters showed that the moisture content of the spent coffee grounds to be processed in the relevant unit for pelletization, the transportation distance the truck will travel to collect the spent coffee grounds and distribute the coffee pellets, and the alternation of the energy supply across Europe also play a significant role in the total environmental impact of the overall process.

The analysis of environmental impacts for spent coffee grounds with moisture contents of 25%, 50% (baseline), and 75% shows that the overall environmental burden heavily depends on the initial moisture level, especially during energy-intensive processing steps. Across nearly all environmental impact categories, including Climate Change, Fine Particulate Matter Formation, and Freshwater Consumption, electricity consumption for drying and pelletizing emerged as the dominant contributor. In the figure below, the normalized total values for all environmental impact categories (of all the processes) are presented to understand their relationship with the raw material’s moisture content. Notably, the normalized data indicate that higher moisture content in SCGs directly correlates with an increased relative contribution from the Electricity for Drying and the Electricity for Pelletizing processes. For instance, in the 50% moisture baseline, Electricity for the drying process accounts for 42.18% of the climate change impact, while at 25% moisture, this drops to 34.40%. This trend underscores that the additional energy required to remove more water from wetter SCGs significantly amplifies the environmental footprint of the coffee pellet production process.

Conversely, a reduction in SCG moisture content, as observed in the 25% moisture scenario, generally mitigates the environmental impacts associated with drying. While this reduction in drying energy lessens the overall impact, it can lead to a proportional shift in the relative importance of other process stages. For example, in the 25% moisture case, the Electricity for Pelletizing shows a slightly higher relative contribution to Climate Change (34.59%) compared to Electricity for Drying (34.40%), suggesting that as drying becomes less impactful, the energy demands of pelletizing become more prominent. Furthermore, transportation impacts and diesel consumption, particularly for the raw SCGs, also exhibit sensitivity to moisture content, as a greater mass of wetter material must be transported to yield the same functional unit of 1 tonne of coffee pellets. These findings highlight that optimizing SCG moisture content is a crucial leverage point for minimizing the environmental footprint of coffee pellet production, primarily by reducing the energy demand for moisture removal.

##### Electricity, alternative grids across Europe

**Fig. 15:**
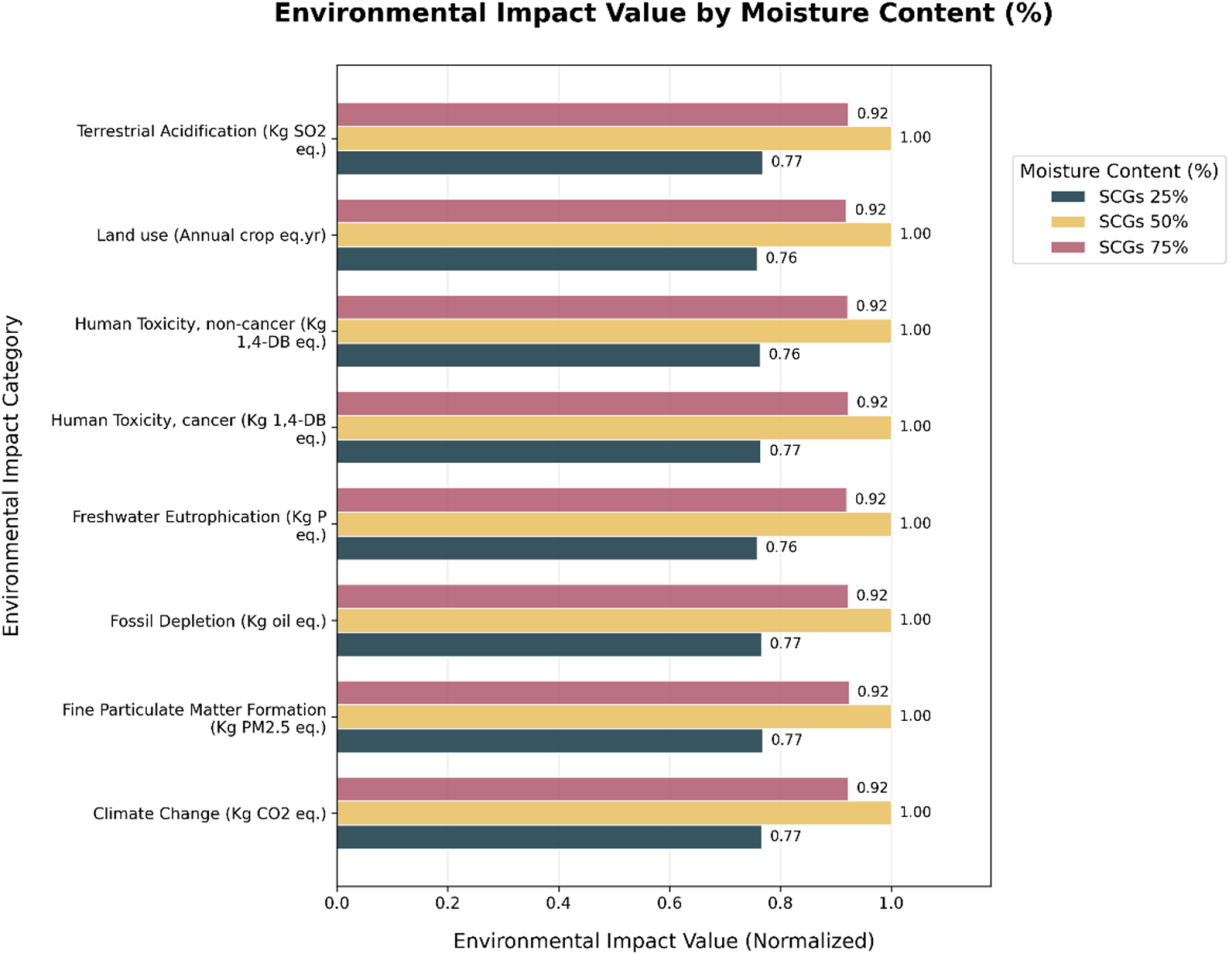
Normalized environmental impacts by SCG moisture content (25%, 50%, 75%) for 1000 kg of coffee pellets. Values are normalized to the highest per category.

The analysis of alternative electricity grids across Europe, including Central and Eastern Europe (Poland), Northern Europe (Sweden), Southern Europe (Greece), and Western Europe (France), reveals that the regional electricity mix significantly influences the overall environmental impact of coffee pellet production. The normalized data, as presented in the following figure (Fig. 15), indicate that the environmental performance of electricity-intensive processes, such as drying and pelletizing, varies considerably depending on the carbon intensity and primary energy sources of the local grid. For instance, reliance on the Central and Eastern European (PL) grid shows that Electricity Drying contributes 41.327% to Climate Change, whereas using the Northern European (SE) grid reduces this contribution to 5.031%. This stark difference highlights the substantial environmental benefits of sourcing electricity from cleaner grids. Conversely, the Southern European (GR) grid results in Electricity Drying contributing 37.642% to Climate Change, demonstrating how reliance on grids with a higher share of fossil fuels can significantly elevate impacts across various categories. This regional variability underscores the importance of geographical location and energy infrastructure in determining the sustainability of industrial processes. The choice of electricity supply, therefore, represents a critical factor in mitigating the environmental burden associated with coffee pellet production, highlighting the potential benefits of sourcing electricity from cleaner grids or investing in on-site renewable energy generation.

##### Transportation distance

**Fig. 16:**
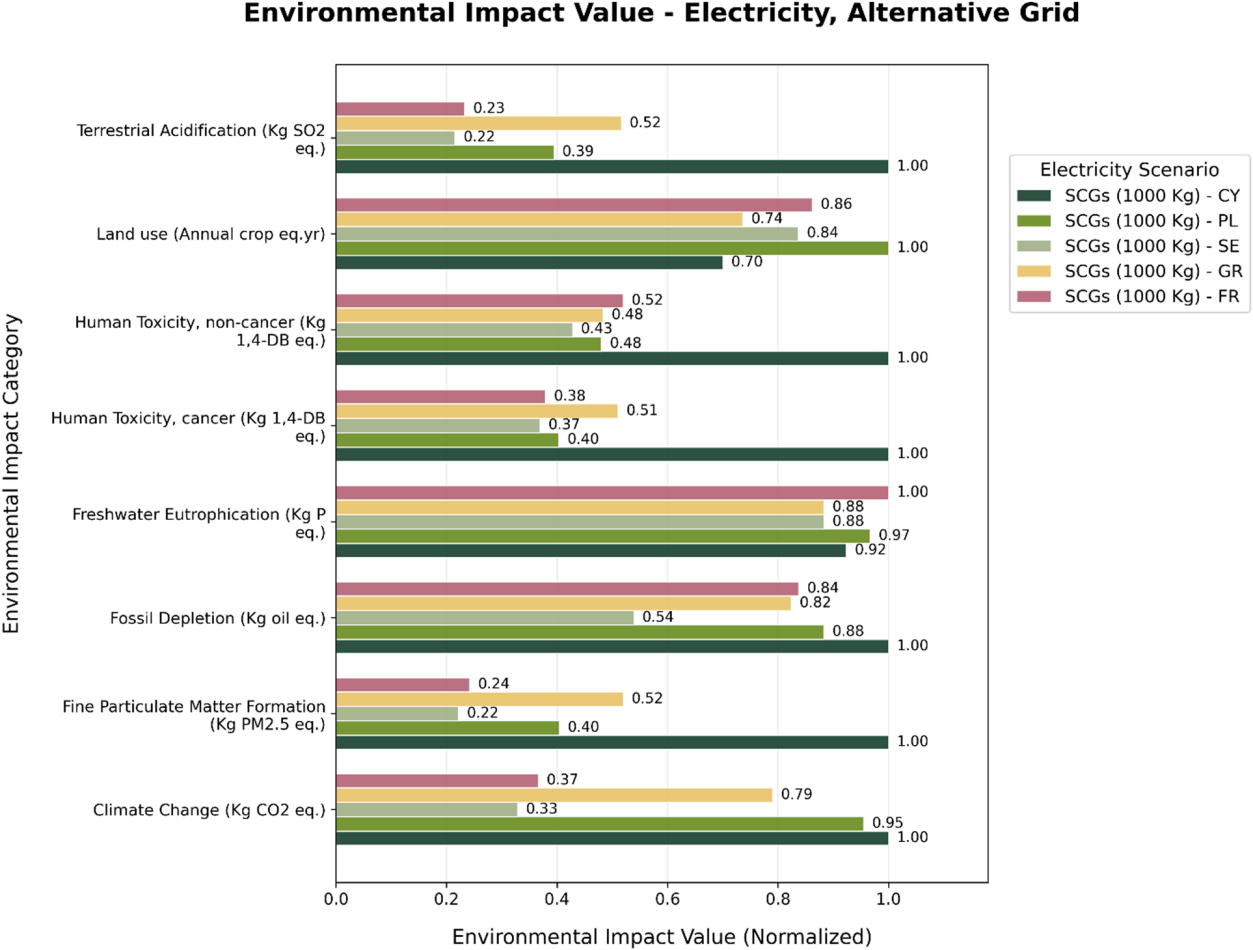
Normalized environmental impacts of electricity (alternative grid) across countries (CY, PL, SE, GR, FR) for 1000 kg of coffee pellets. Values are normalized to the highest per category.

The data, presented in the Fig. 16 in a normalized format, compares the environmental impact values for SCGs transported over 100 km (baseline), 80 km, and 120 km across various categories such as Climate Change, Fine Particulate Matter Formation, Fossil Depletion, Freshwater Eutrophication, Human Toxicity (cancer and non-cancer), Land Use, and Terrestrial Acidification. For most categories, a shorter transportation distance of 80 km results in lower environmental impact values compared to the 100 km baseline, while a longer distance of 120 km shows higher impacts. For example, Climate Change (Kg CO2 eq.) is 0.89 for 80 km, 0.94 for 100 km, and 1.00 for 120 km, indicating a direct correlation between transportation distance and environmental burden.

This analysis underscores the critical role of transportation logistics in the overall environmental footprint of SCG pellet production. Minimizing transportation distances for both the collection of waste coffee grounds and the distribution of coffee pellets can significantly reduce environmental impacts across multiple categories. The normalized values clearly demonstrate that even relatively small changes in transportation distance (e.g., a 20 km reduction from the baseline) can yield measurable environmental benefits. Therefore, optimizing collection and distribution routes and establishing processing facilities closer to coffee waste sources are crucial strategies for enhancing the environmental sustainability of the coffee pellet production process.

##### Cost-benefit analysis

**Fig. 17:**
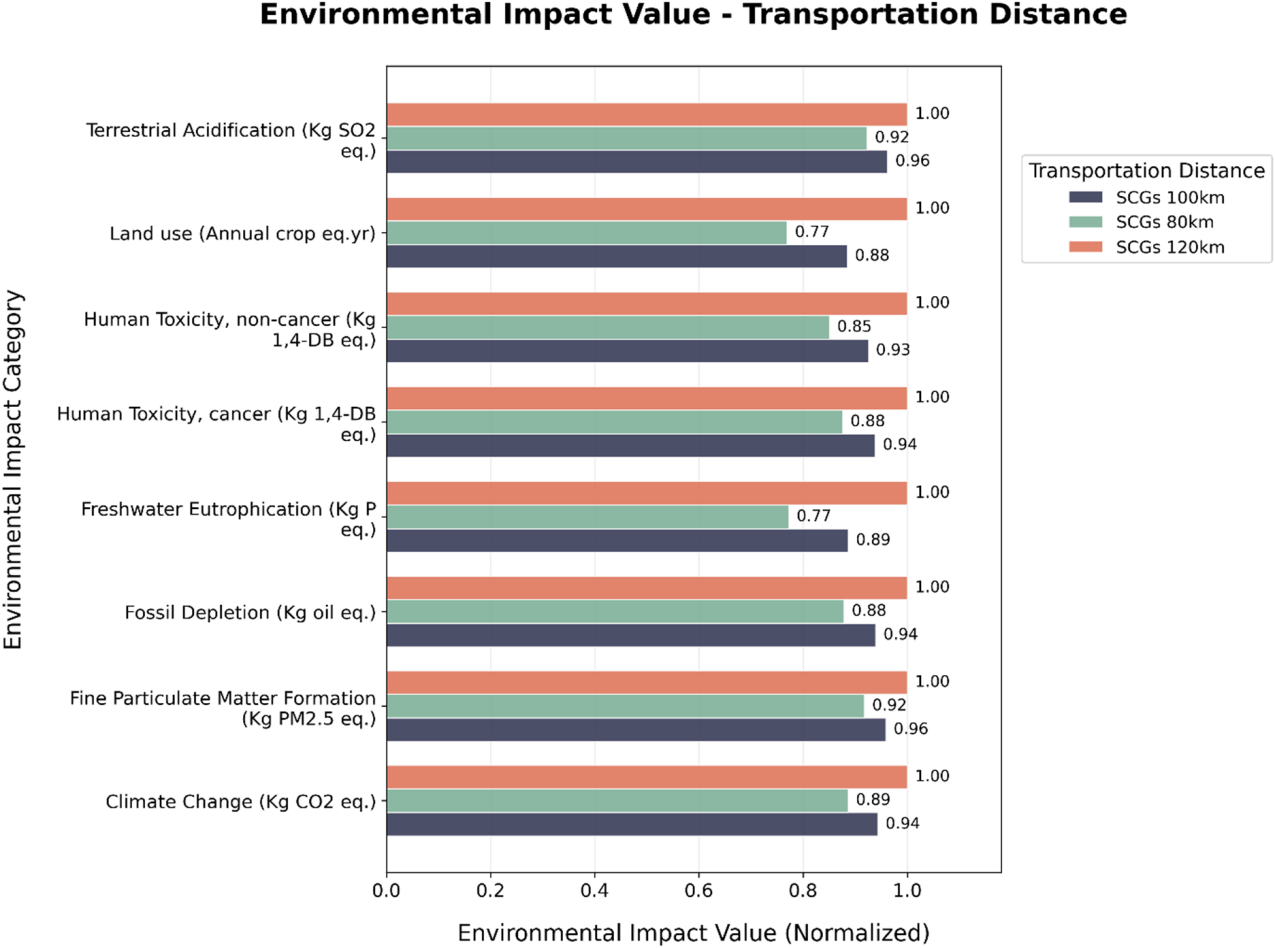
Normalized environmental impacts of transportation distance impact. Values are normalized to the highest per category.

The preliminary techno-economic analysis of the project, as explained in the Fig. 17, focuses on the capital expenditure (CAPEX) and operational expenditure (OPEX) associated with transforming spent coffee grounds into pellets. The CAPEX primarily includes the transportation system (trucks, bins/containers, handling equipment) for collecting waste coffee grounds and distributing pellets, as well as the pelletization plant equipment (feeding tank, drying unit, pelletizing unit, cooling unit, and packaging unit). The OPEX covers fuel for transportation, driver labor, truck maintenance, transport operations, electricity consumption for the plant (drying, pelletizing, cooling units), thermal energy for the drying unit, labor (operators, supervisory staff, administrative), equipment maintenance and spare parts, rent/facility costs, insurance, and general overheads. This analysis provides a foundational understanding of the financial investment and recurring costs involved in establishing and running such a facility.

**Fig. 18:**
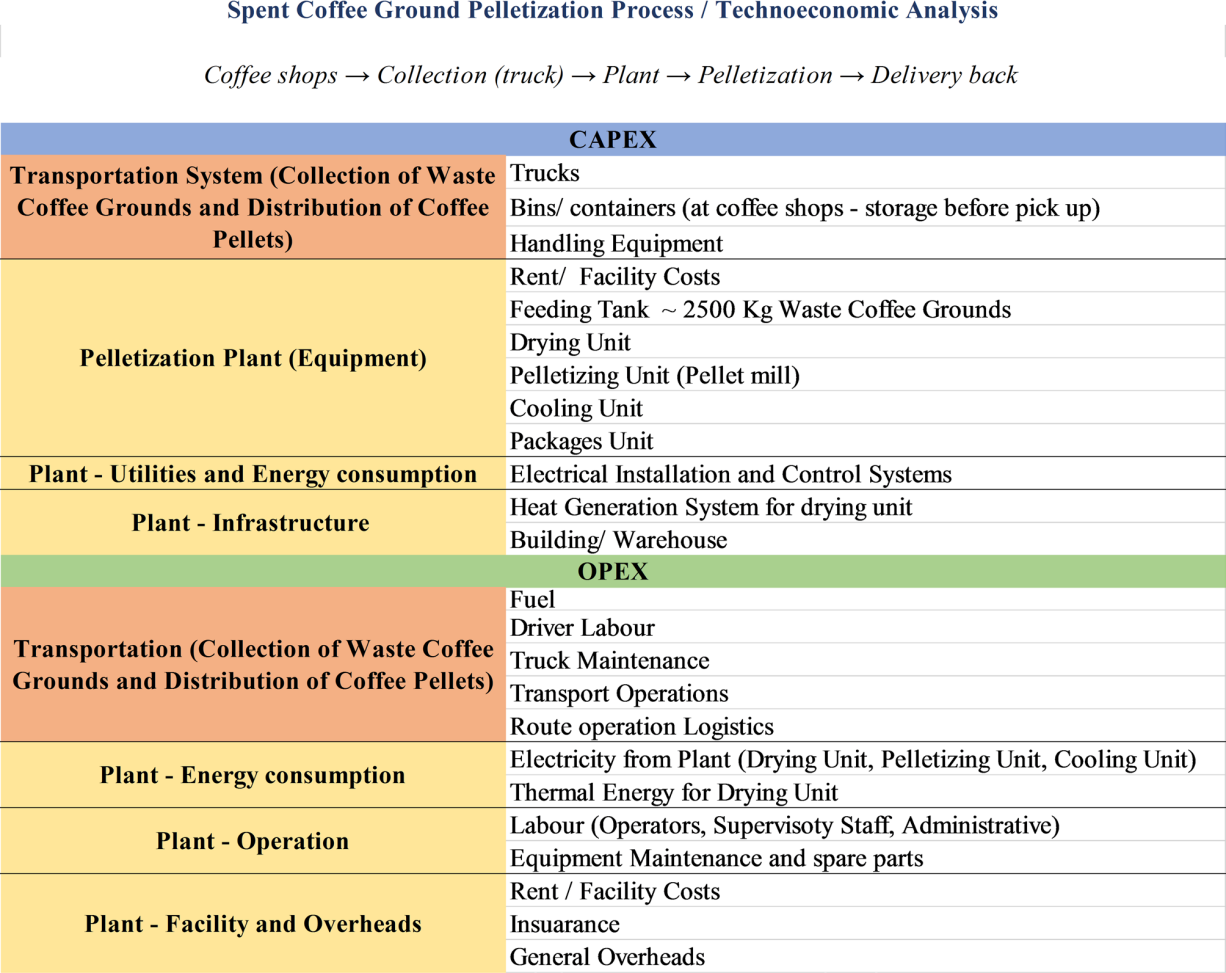
Structure of the techno-economic analysis for the spent coffee grounds pelletization process, outlining CAPEX and OPEX components across transportation and plant operations.

It is important to note that this is a preliminary techno-economic analysis and, as such, does not encompass all aspects of a full techno-economic assessment. Specifically, the scope of this research does not include the incorporation of the Cyprus (Cy) inflation rate or a sensitivity analysis of ± 2% on the models, which would typically be considered in a more comprehensive study. These additional factors would provide a more robust understanding of the project’s financial viability under varying economic conditions and market fluctuations. However, the current analysis successfully identifies the key cost drivers and operational considerations for the coffee pellet production process (Fig. 18).

.

## Conclusions

This study demonstrates the feasibility of converting spent coffee grounds (SCGs) into solid biofuel pellets through a combination of experimental analysis and life cycle assessment (LCA). Thermochemical characterization confirmed that SCG pellets exhibit a high calorific value and acceptable ash content, meeting key performance thresholds defined by EN ISO 17225-6. However, the elevated moisture content of the raw feedstock remains a technical limitation, requiring pre-drying to ensure efficient combustion and stable storage.

The LCA results identify drying and transportation as the dominant contributors to environmental impacts, primarily due to electricity consumption and diesel use. Among the examined scenarios, the combined sun drying and photovoltaic electricity configuration yields the lowest impacts across all assessed categories, including Global Warming Potential, Acidification Potential, Eutrophication Potential, and Fossil Fuel Depletion. Differences in Ozone Depletion Potential were limited, indicating lower sensitivity of this category to the evaluated energy substitutions.

Sensitivity analysis further highlights the influence of key parameters on environmental performance. Moisture content significantly affects process efficiency by directly influencing drying requirements, whereas electricity supply depends strongly on the carbon intensity of regional grid mixes. Transportation distance also plays a critical role, with shorter distances leading to reduced environmental burdens. These findings underline the importance of balanced moisture levels, cleaner energy sources, and optimized logistics in minimizing impacts. A preliminary cost–benefit analysis indicates that integrating renewable energy can improve economic performance by reducing operational energy costs, despite higher initial investment requirements. This assessment remains indicative and will be expanded into a detailed techno-economic analysis following the finalization of the production system modelling.

Overall, the results support integrating SCG pelletization into circular bioeconomy strategies, particularly in regions with high solar availability. The approach enables the valorization of an abundant waste stream while contributing to resource efficiency and emission reduction. Future research should investigate alternative system configurations, including centralized and decentralized processing models, further optimize drying processes, and validate under real operating conditions.

## Data Availability

The datasets generated and/or analysed during the current study are available from the corresponding author upon reasonable request.
